# Voxel-based, brain-wide association study of aberrant functional connectivity in schizophrenia implicates thalamocortical circuitry

**DOI:** 10.1038/npjschz.2015.16

**Published:** 2015-05-06

**Authors:** Wei Cheng, Lena Palaniyappan, Mingli Li, Keith M Kendrick, Jie Zhang, Qiang Luo, Zening Liu, Rongjun Yu, Wei Deng, Qiang Wang, Xiaohong Ma, Wanjun Guo, Susan Francis, Peter Liddle, Andrew R Mayer, Gunter Schumann, Tao Li, Jianfeng Feng

**Affiliations:** 1 Centre for Computational Systems Biology, Fudan University, Shanghai, China; 2 Centre for Translational Neuroimaging, Division of Psychiatry and Applied Psychology, Institute of Mental Health, University of Nottingham, Nottingham, UK; 3 The Mental Health Center and the Psychiatric Laboratory, West China Hospital, Sichuan University, Chengdu, China; 4 Key Laboratory for Neuroinformation, Ministry of Education of China, School of Life Science and Technology, University of Electronic Science and Technology of China, Chengdu, China; 5 Institute of Mental Health, Second Xiangya Hospital, Central South University, Changsha, China; 6 School of Psychology and Center for Studies of Psychological Application, South China Normal University, Guangzhou, China; 7 Sir Peter Mansfield MR Centre, University of Nottingham, Nottingham, UK; 8 The Mind Research Network, Albuquerque, USA; 9 Medical Research Council—Social, Genetic and Developmental Psychiatry Centre, Institute of Psychiatry, King’s College London, De Crespigny Park, London, UK; 10 Department of Computer Science, University of Warwick, Coventry, UK; 11 School of Life Science and the Collaborative Innovation Center for Brain Science, Fudan University, Shanghai, China

## Abstract

**Background:**

Wernicke’s concept of ‘sejunction’ or aberrant associations among specialized brain regions is one of the earliest hypotheses attempting to explain the myriad of symptoms in psychotic disorders. Unbiased data mining of all possible brain-wide connections in large data sets is an essential first step in localizing these aberrant circuits.

**Methods:**

We analyzed functional connectivity using the largest resting-state neuroimaging data set reported to date in the schizophrenia literature (415 patients vs. 405 controls from UK, USA, Taiwan, and China). An exhaustive brain-wide association study at both regional and voxel-based levels enabled a continuous data-driven discovery of the key aberrant circuits in schizophrenia.

**Results:**

Results identify the thalamus as the key hub for altered functional networks in patients. Increased thalamus–primary somatosensory cortex connectivity was the most significant aberration in schizophrenia (*P*=10^−18^). Overall, a number of thalamic links with motor and sensory cortical regions showed increased connectivity in schizophrenia, whereas thalamo–frontal connectivity was weakened. Network changes were correlated with symptom severity and illness duration, and support vector machine analysis revealed discrimination accuracies of 73.53–80.92%.

**Conclusions:**

Widespread alterations in resting-state thalamocortical functional connectivity is likely to be a core feature of schizophrenia that contributes to the extensive sensory, motor, cognitive, and emotional impairments in this disorder. Changes in this schizophrenia-associated network could be a reliable mechanistic index to discriminate patients from healthy controls.

## Introduction

One of the earliest mechanistic notions proposed to account for the myriad of symptoms seen in individuals with psychotic disorders is the concept of ‘sejunction’ (fragmentation of localizable association links) put forward by Wernicke in 1894.^1^ Wernicke believed that a disjunction between distinct functional modules that involve both sensorimotor and association areas of the brain generate symptoms of psychosis.^2^ The notion of sejunction has given rise to the current concept of dysconnectivity in schizophrenia,^3,4^ resulting in numerous attempts to locate the hypothesized aberrant networks. Extant neuroimaging studies have highlighted a number of abnormal regional connections in patients contributing to the general acceptance of a vaguely defined ‘widespread dysconnectivity’. However, the putative ‘sejunction circuitry’—the most consistent and characteristic aberration in functional connections that define the syndrome of schizophrenia—is still elusive as no constant patterns that reliably explain schizophrenia’s complex and heterogeneous symptoms have emerged to date. This poses a challenge to the reliability of schizophrenia as a diagnostic entity.

Notwithstanding its narrow diagnostic definition, schizophrenia is remarkably heterogeneous in outcome, and widespread variable patterns of changes in neural networks can contribute to its varied clinical expressions. When searching for final common pathways in any multifactorial complex disease, it is imperative that the noise induced by heterogeneity is tackled by increasing sample size.^5^ Unfortunately, most studies investigating dysconnectivity in schizophrenia have modest sample sizes, often insufficient for observations to survive even lenient corrections for multiple comparisons.^4^ To some extent, validation of results from small samples can be achieved through a meta-analytic approach. For example, consistent structural changes in the anterior insula and anterior cingulate cortex have emerged when morphometric data from more than 2,500 patients were synthesized by several groups.^6–8^ Unfortunately, the two common methods used for studying functional connectivity—seed region approach and independent component analysis—do not allow unbiased pooling of results reported in individual studies. Seed-based analysis with *a priori* specification of brain regions is not a ‘discovery approach’, but allows for testing hypothesis that are cultivated by prior observations. The independent component analysis approach, though a data-driven approach well suited for novel discoveries of aberrant connectivity, is only partially independent of prior assumptions,^9–11^ as the components are assumed to arise from statistically independent sources and are often selected on the basis of prior expectations of plausible signals, e.g., large-scale networks such as the default mode network. As a result, across studies, even similarly named components have a diverse anatomical distribution that again precludes pooled synthesis of individual studies.

The research into the pathophysiology of schizophrenia in general, and functional neuroimaging in particular, is plagued by partial replications and lack of convincing refutations. A summary of systematically selected resting-state connectivity studies on schizophrenia provided by Patterson-Yeo *et al.* ^4^ highlight this issue (an update of this summary is provided in Supplementary Table S1). There is an urgent need to use methods that will allow large-scale pooling of data to both reduce the impact of heterogeneity and allow the study of stratified subgroups. To provide both greater confidence and accuracy in identifying which specific regions and their functional connections contribute most to schizophrenia, it is important to be able to use a voxel-based brain-wide analysis strategy, in addition to region-based. In this paper, we therefore report the first meta-study using both a region and voxel-based unbiased brain-wide association study (BWAS) approach on resting-state functional magnetic resonance imaging (MRI) data. Three resting-state schizophrenia data sets from China, USA, and the UK comprising of 415 patients and 405 matched controls were collated to produce the largest resting-state study in schizophrenia. The BWAS is modeled along the lines of the genome-wide associate study (GWAS) where large genetic data sets are pooled to identify significant genetic variations in specific disorders. The data-driven GWAS approach has contributed to firmly refuting a major role for certain genetic loci derived from small studies. The BWAS approach used here also uses a higher-order community discovery algorithm to account for the inherent modular nature of brain networks.

In addition, we investigate clinical associations among the so-called ‘sejunction circuitry’ (i.e., the most consistent abnormal pattern of connectivity discovered from the whole-brain data-driven search), symptom severity, and illness duration, and the extent to which it can reliably discriminate between patients and controls using a pattern classification approach.

## Materials and Methods

### Participants

Participants’ demographic and clinical characteristics are summarized in Table 1. More details of subjects and the collection of these data are provided in the Supplementary Information section.

### Image acquisition and preprocessing

All the Taiwan, COBRE, and Xiangya imaging data were acquired using a 3-T Siemens Trio Tim MRI scanner with an eight or a twelve channel phased array head coil, whereas that from Huaxi data were acquired using a 3-T General Electric MRI scanner. The Nottingham data set was acquired using a 3-T Philips Achieva MRI scanner. During data acquisition, the subjects were instructed to keep their eyes closed but not fall asleep (Huaxi, Taiwan, and Xiangya sites) or to remain with their eyes open staring at a fixation cross (COBRE and Nottingham sites). Image preprocessing steps included slice timing, within-subject realignment, spatial normalization to the stereotactic space of the Montreal Neurological Institute with voxel size of 3×3×3 mm^3^, linear detrending, band-pass filtering (0·01~0·08 Hz), and scrubbing. In all cases the data were smoothed spatially (FWHM 8 mm) and nuisance signal were regressed (including six motion parameters, the global, white matter, and cerebrospinal fluid signals). Any data affected by head motion (maximal motion between volumes in each direction, and rotation about each axis) of >3 mm or rotation of >3° was excluded. The Supplementary Information provides additional details of the imaging acquisition and data preprocessing.

### Voxel-wise and atlas-based brain-wide association studies

Step 1: analysis within each imaging center.

In the present study, each resting-state fMRI image included 47,636 voxels, which is based on the automated anatomical labeling atlas. For each pair of voxels, the time series were extracted and their correlation was calculated for each subject followed by z-transformation. Two-tailed, two-sample *t*-tests were performed on the 1,134,570,430 (47,636×47,635/2) Fisher’s *z*-transformed correlation coefficients to identify significantly altered functional links in schizophrenia patients compared with controls within each imaging center. The effect of age, gender ratios, antipsychotic dose, and head motion were regressed within each data set in this step.

Step 2: combination of results from all imaging centers.

The Liptak–Stouffer *z*-score method,^12^ which is a well-validated method for multisite data sets and has previously been used widely in multisite gene^13–16^ and MRI data analysis^7,17^ was then used to combine the results from the individual data sets. Specifically, the *P* value of each functional connectivity result from two-sample *t*-test in step 1 was converted to its corresponding *z*-score. This was calculated first as in equation: *z*_*i*_=*Φ* ^−1^(1−*p_i_*), where *Φ* is the standard normal cumulative distribution function and *i* represent the *i* site. Next, a combined *z*-score for a functional connectivity was calculated using the Liptak–Stouffer formula:

$$\;Z=\frac{\sum_{i=1}^{k}w_{i}z_{i}}{\sqrt{\sum_{i=1}^{k}w_{i}^{2}}},$$

which follows a standard normal distribution under the null hypothesis; where $w_{i}=\sqrt{\text{sample size}}$ is the weight of the *i* data set. Finally, The *Z* is transformed into its corresponding *P* value and a Bonferroni procedure was used to correct for multiple comparisons.

Step 3: calculating a measure for the association.

A measure for the association (MA) between a voxel *i* and the brain disorder was then defined as: MA=*N*_*α*_, where *N*_*α*_ is the number of links between voxel *i* and every other voxel in the brain that have a *P* value of less than *α* (in the present study *α*=0.05/(47,363×47,635/2)) in *t*-tests. A larger value of *MA* implies a more significant alteration in functional connectivity.

To verify the voxel-wise results, we also parcellated the whole brain into 90 regions of interest according to the automated anatomical labeling atlas.^18^ The names of the regions of interest and their corresponding abbreviations and anatomical classification^19,20^ are listed in Table 2. The functional connectivity was evaluated between each pair of regions using a Pearson correlation coefficient.

Consistency of effect-size across studies was examined using the Cochran’s Q test.^21^ Between-study (effect) heterogeneity was indicated by a Q statistic *P*<0.05. The links with significant heterogeneity were additionally assessed using random-effects model.^22^

### Clinical correlates

We investigated whether the emerging pattern of dysconnectivity correlated with clinical variables (positive and negative syndrome scale, PANSS and illness duration) using partial correlation. The data without PANSS score (Nottingham) was excluded in our analysis. Specifically, we calculated the partial correlation between the strength of each altered functional connection and PANSS score and illness duration after removing the effect of sites. To further test the clinical correlates of the ‘sejunction’ concept in classification of schizophrenia, we applied a support vector machine^23^ approach using the altered pairwise associations from BWAS to discriminate patients from controls. We used a leave-one-out cross-validation strategy to estimate the generalizability of this classifier and estimated accuracy, sensitivity, and specificity. Permutation tests were used to estimate the statistical significance of the observed classification accuracy.

## Results

### Voxel-based BWAS

**Figure 1:**
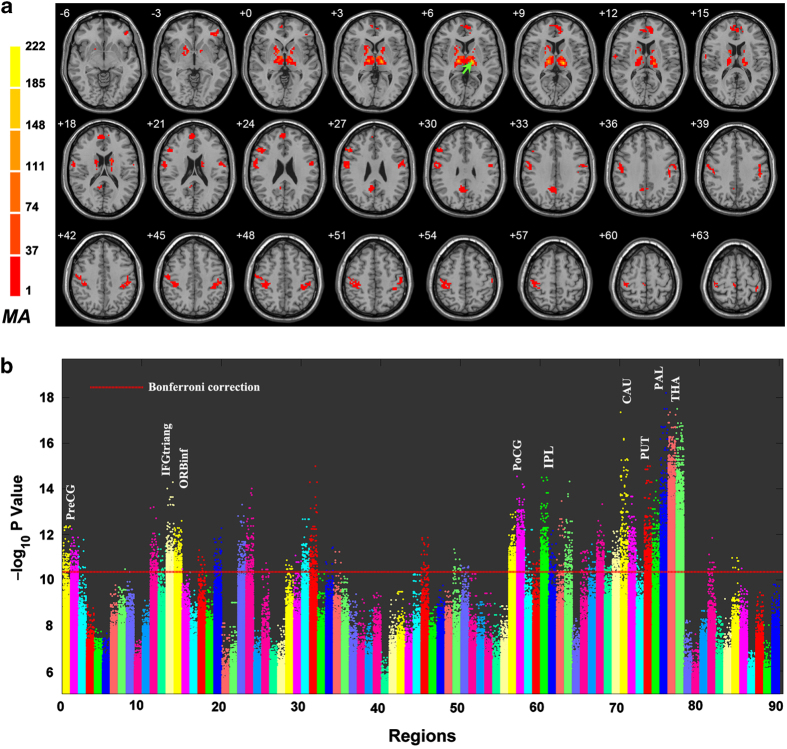
Anatomical location of consistent aberrant connectivity in schizophrenia obtained from voxel-based BWAS. (**a**) Voxels showing the largest number of whole-brain connectivity aberrations in patients with schizophrenia (cluster size >20). Color bar represents the measure of association (MA) given by the number of significantly affected links relating to each voxel. Supplementary Table S2 describes the clusters in more detail. (**b**) Manhattan plot of voxel-based BWAS results with voxels grouped in accordance with AAL atlas labels. Right and left homologous regions are placed adjacent to each other. Each dot represents a voxel-based pairwise connection. Note that there are a total of 47,636×47,635/2 links and we only plot *P*<10^−5^. The red dotted line is the Bonferroni correction threshold 4.4×10^−11^. Thalamus (regions 77 and 78) includes the maximal number of voxels showing aberrant connectivity. Other notable regions are also labeled. For abbreviated labels, refer to Table 2. Results shown are obtained using a meta-analytical approach correcting for inter-site variation in effect-size, age, gender ratios as well as antipsychotic dose. AAL, automated anatomical labeling; BWAS, brain-wide association study.

As shown in Figure 1b, the region with the highest number of abnormal functional connections in schizophrenia patients is the thalamus, followed by the postcentral gyrus (PoCG). The smallest *P* value is around 10^−18^, with a total of 9,044 altered links being significant after Bonferroni correction (*P*<0.05, the significance level uncorrected had to be *P*<5.4×10^−7^). The most significantly altered cluster was in the thalamus (Figure 1a: Peak Montreal Neurological Institute coordinate (−12, −18, 6), cluster size 442, MA 222) and involved 5,620 links, while the second most significantly altered cluster was in the postcentral gyrus (Peak Montreal Neurological Institute coordinate (−48, −27, 42), cluster size 167, *MA* 61) and involved 1,045 links. Other clusters primarily involve subcortical regions involved in brain reward processing, including caudate, pallidum, and putamen and frontal regions including superior medial frontal gyrus, inferior frontal gyrus (triangular), and middle frontal gyrus. The coordinates of the significant clusters are summarized in Supplementary Table S2.

### Atlas-based BWAS

**Figure 2:**
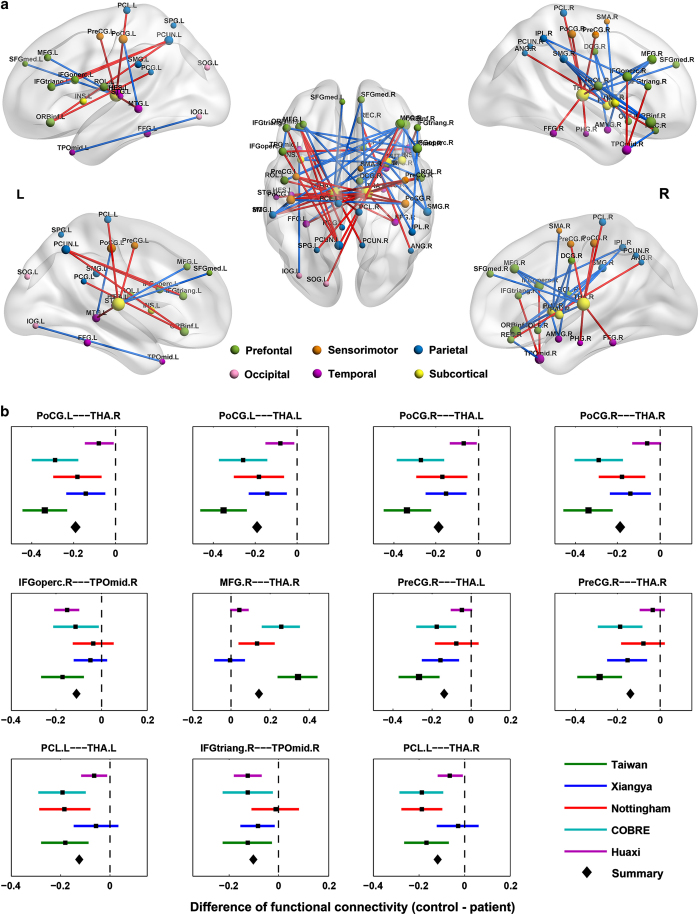
A schematic diagram showing AAL-based connectivity differences between the schizophrenia and the control group. (**a**) Links with increased associations in patients are shown in red, decreased associations are in blue (*P*<0.05, Bonferroni connection). Different colors of nodes correspond to major divisions based on the functional anatomy of the brain. The glass brains were generated using BrainNet Viewer (http://www.nitrc.org/projects/bnv/). (**b**) Forest plots showing a meta-analysis of the association of the significant functional connectivities (top 11 links) with schizophrenia. We can see that the altered patterns of functional connections were consistent across the data sets from the five different imaging centers. AAL, automated anatomical labeling; L, left; R, right.

Using the automated anatomical labeling template, the most significantly changed region was again the thalamus: with a total of 33 changed links out of 74 links (Figure 2 and Supplementary Table S3, Bonferroni correction *P*<0.05). The region-wise results are therefore consistent with the voxel-based meta-analysis. In the atlas-based ‘sejunction map’ plotted in Figure 2, one main hub is involved: the thalamus. Another notable feature is that the strength of functional links between the thalamus and the frontal cortical regions are mostly reduced in patients, other than those involving the motor and temporal cortical regions that are increased (Figure 2). Moreover, the altered patterns of functional connections are very consistent across the data sets from the three different centers, despite the differences in the clinical populations: the mean functional links all increase or decrease in the same direction when comparing schizophrenia patients with healthy controls. More than 70% of altered links showed a consistent pattern; among the 29.7% links showing across-sites variation, the effect was due to a single center. Figure 2b show the altered patterns of top 11 functional connections that were most consistent across the data sets from the five sites. Another notable feature is that most changes involve links between the major functional divisions/lobes of the brain rather than within them, e.g., aberrant connectivity between subcortical regions and prefrontal or motor cortices, rather than among subcortical regions themselves.

### Clinical correlates of the altered circuit

Studying the correlation between thalamocortical connections and clinical variables (PANSS scores and illness duration) at a false discovery rate-corrected *P*<0.05, we noted several significant relationships. In general, lower thalamofrontal connectivity predicted higher burden of positive symptoms; higher thalamosensory (PoCG, STG) connectivity predicted higher burden of negative symptoms (Table 3). Interestingly, none of the altered nonthalamic links predicted any of the clinical variables tested (Supplementary Table S4) at the corrected statistical threshold, indicating the primacy of thalamic aberrations in schizophrenia. Furthermore, we also performed correlation analyses between altered functional connectivities and all PANSS subscores and the result is summarized in Supplementary Figure S1. Interestingly, the thalamus–PoCG and thalamus–PCG functional connectivities were most strongly associated with difficulty in abstract thinking and thalamus–superior frontal gyrus connections with delusions.

**Figure 3:**
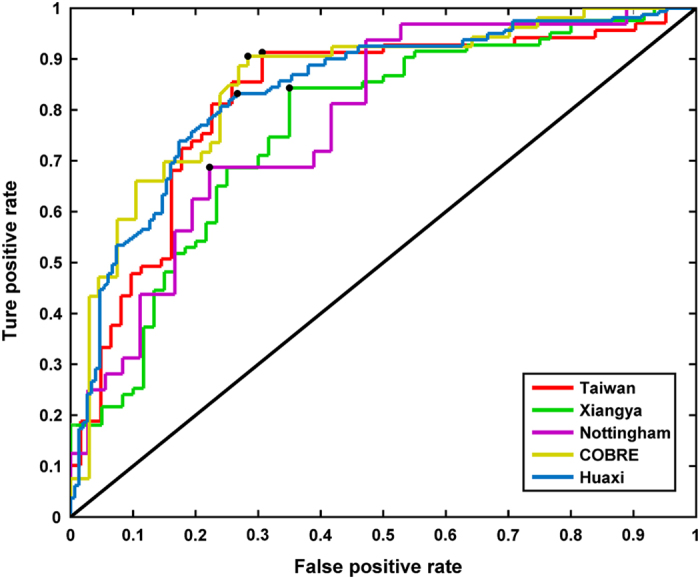
ROC curve derived from the aberrant connectivity patterns emerging from the brain-wide association study using regional parcellations. Using the connectivity metrics as discriminant markers in a multivariate pattern classification framework, the true-positive rate is plotted against the false-positive rate. The black dots denote the best cut-off values. ROC, receiver operating characteristic.

The support vector machine analysis (Figure 3) indicated a high degree of accuracy in discriminating patients and controls on the basis of the aberrations in connectivity identified from the atlas-based BWAS (overall accuracy from all data sets pooled=75.81%). The accuracy rates for distinguishing patients from healthy controls varied within a small range (73.5 to 80.9%, i.e.,7.4%) across the data sets. Permutation tests revealed that within and across data sets, discrimination accuracies were highly significant (*P*<0.01 in all cases). Support vector machine results are summarized in Supplementary Table S5.

## Discussion

We have presented a large systematic investigation of altered brain-wide functional connectivity in schizophrenia. To our knowledge, this is the first time that both a regional and voxel-based analysis that covers the entire brain with no *a priori* selections have been conducted in such a large multisite sample. The results have revealed highly replicable aberrations in connectivity involving thalamocortical connections. Importantly, the putative ‘sejunction’ that is mostly anchored upon thalamus varies with symptom severity as well as illness duration and provides a good discrimination accuracy between patients and controls (73.5–80.9%), despite variations in the data acquisition across several independent sites. This emphasizes the reliability of resting fMRI as an investigative tool to study the pathophysiology of heterogeneous syndromes such as schizophrenia.

Many previous studies investigating brain changes associated with schizophrenia have mainly been based on seed-based or independent component analysis approaches. Although these studies have suggested the importance of anomalies in the default mode network^24,25^ and other intrinsic networks,^26–28^ both resting-state and task-related studies have reported varying findings that has not converged on a single pattern. Nevertheless, extant literature on functional connectivity in schizophrenia points towards increased default mode network and decreased prefrontal connectivity,^29^ but significantly biased by the excessive focus on default mode network for resting fMRI and prefrontal cortex for task-based studies.^4^ Although the findings from each of these studies are likely to be potentially crucial to the pathophysiology of schizophrenia, our rigorous data-driven discovery approach using a very large sample suggests that the most robust pattern within the ‘widespread dysconnectivity’ involves the thalamocortical circuitry in schizophrenia.

Several fMRI findings support the idea that the thalamus is a key region in schizophrenia,^29–34^ but all of these studies focus on thalamus only, thus failing to demonstrate the prominence of thalamic connections among the ubiquitous dysconnectivity seen in patients. Our finding is essentially a strong and data-driven verification of thalamic–cortical dysconnectivity in schizophrenia. Across two different approaches (voxel-wise versus atlas-based) and across five data sets, we not only show that a large effect pertaining to aberrant connectivity of the thalamus, but we also show that the abnormalities involving this structure is more prominent than all other large-scale connectivity aberrations in schizophrenia.

A distinct feature of the identified subcortical (thalamic) connections is that there is a pattern of frontal reduction and sensorimotor enhancement (PreCG, LING, FFG, PCL, and PoCG) in connectivity. This pattern was first reported in a study that included 62 subjects with schizophrenia^29^ and has been replicated and shown to be a common feature across the two major psychotic disorders—bipolar disorder and schizophrenia using another seed-based fMRI analyses^32^ although these samples were not sufficiently powered to detect correlations with specific clinical symptom scores. Our findings confirm that this apparently hierarchical distribution of thalamocortical abnormalities^32^ is related to symptom burden in schizophrenia. This observation raises an interesting question of whether the failure to update/propagate prediction models upon processing sensory information^35,36^ could be mapped on to the frontal reduction and sensorimotor enhancement pattern of thalamic dysconnectivity in patients.

Experimental neuroimaging using magnetic stimulation has provided more direct evidence of thalamic dysconnectivity in schizophrenia.^37^ Given the normally high degree of connectivity of thalamus within the brain, we cannot dismiss the possibility that thalamic aberrations are nonspecific in schizophrenia, and only reflect the generalized nature of network-level aberrations that arise in this illness. Nevertheless, the pattern of selective increase in certain thalamic connections while the others show a reduction suggests a putative illness-related imbalance. As highlighted by earlier authors,^29^ this pattern is also consistent with the importance of this structure in developmental cortical maturation and the neurodevelopmental theories pertaining to schizophrenia.

Our analysis of correlations between the thalamo–frontal and thalamo–sensorimotor functional connectivities revealed a restricted pattern of associations with specific symptoms. Reduced functional connections between the thalamus and superior frontal gyrus were particularly associated with PANSS delusion scores, in agreement with a previous structural-based study.^38^ On the other hand, increased thalamic functional connections with both the pre- and postcentral gyri were particularly associated with difficulty in abstract thinking (see Supplementary Figure S1). This latter finding may reflect a role for these aberrant connections in cenesthopathy and perhaps some disturbed aspects of embodied cognition in schizophrenia,^39^ with the sensorimotor system contributing to processing abstract words and conceptual knowledge.^40,41^

Several strengths and limitations must be borne in mind while interpreting this study. We examined a large multisite data set, with a meta-analytic approach to address inter-site variations. More commonly used validation approaches with multiple data sets test whether the same observation survives a type 1 error threshold across the data sets, but ignore the distribution of effect-sizes across the samples. Our meta-analytic approach considers the effect-size distribution to be of more importance than whether observations survive arbitrary thresholds set for data sets of varying sample sizes. In addition to voxel-wise search, we also used an anatomical parcellation scheme to obtain functional links and show a convergence. With regard to limitations, in line with a number of other studies, we used a correlation-based approach to infer brain connectivity; no causal influences can be assumed between the linked regions. Although including medication dose has been covaried when computing functional connectivity, we cannot rule out some possible nonlinear effects and effects due to lifetime exposure. Many of our patients were medicated and antipsychotic medications may attenuate functional connectivity patterns in the short term, though to date it is unclear how thalamocortical connectivity is affected by antipsychotics.^42,43^ The observed dysconnectivity was consistent across sites, despite some samples being antipsychotic-naive (Huaxi), mixed (Xiangya), and mostly medicated (COBRE, Nottingham), suggesting that the effect of antipsychotics on the ‘core’ dysconnectivity in schizophrenia, if present, is weak. In addition to antipsychotics, we addressed other potential confounds including movement artefacts carefully (Supplementary Material).

In summary, we identified the probable ‘sejunction circuitry’ located on thalamic hubs in the largest reported sample of patients with schizophrenia to date. The implications of this finding are three-fold: First, by using a meta-analytic approach on readily available, anatomically parcellated resting-state data, we have provided an initial database upon which further studies could be pooled to continuously shape the search for the consistently dysconnected neural system in schizophrenia. While we do not claim that the frontal reduction and sensorimotor enhancement pattern of thalamocortical dysconnectivity is the core abnormality in schizophrenia, at a more modest level, we suggest that the BWAS approach can facilitate discovery akin to the GWAS, with a greater promise of success due to the proximity of neuronal changes to disease expression (as evidenced by the relationship between dysconnectivity and symptom burden). In addition to the large-scale genomic and enviromic data, such connectomic approaches should finally contribute to our understanding of mechanistic pathways that relate to the expression of the schizophrenia phenotype. Second, our data-driven results challenge some of the existing but inconsistent notions of dysconnectivity, highlighting the importance of an exhaustive bottom-up data mining in the investigation of schizophrenia. Finally, given the potential importance of thalamocortical connectivity to animal models and cognition in schizophrenia, our findings emphasize the role of agnostic data-driven neuroimaging in the pathways of drug discovery. The quest for the core mechanisms of schizophrenia has a long history; our observation clarifies the paths that are likely to be promising in this trail.

## Figures and Tables

**Table 1:** A summary of the demographic information and the psychiatric diagnosis in the present study

*Sites*	*Group*	*Age (years)*	*Sex (male/female)*	*Handedness (right/left)*	*Education (years)*	*Positive scale*	*Negative scale*	*General scale*	*Duration of illness*
Taiwan	Healthy	29.87±8.62	25/37	61/1	15.29±2.39	—	—	—	—
	Patient	31.59±9.60	35/34	68/1	14.19±2.16	11.92±4.71	13.61±6.33	27.28±9.64	7.17±6.61
Xiangya	Healthy	27.17±6.64	35/25	—	13.51±3.16	—	—	—	—
	Patient	23.37±7.83	49/34	—	13.22±2.77	19.84±6.31	21.56±7.66	39.24±11.75	1.38±1.35
Nottingham	Healthy	33.38±8.98	26/10	32/4	—	—	—	—	—
	Patient	33.34±9.05	27/5	27/5	—	3.84±3.18	3.13±3.63	5.94±3.89	8.91±6.94
COBRE	Healthy	34.82±11.28	46/21	65/2	14.00±1.78	—	—	—	—
	Patient	36.75±13.68	42/11	42/11	13.20±1.83	14.84±4.53	14.42±4.97	29.88±8.27	—
Huaxi	Healthy	27.80±12.50	95/85	180/0	13.08±3.30	—	—	—	—
	Patient	24.31±8.05	90/88	178/0	11.91±3.30	24.48±6.05	19.68±7.67	46.70±8.87	0.99±2.13

**Table 2:** The names, abbreviations (Abbr.), and anatomical classification^19,20^ of the regions of interest

*No.*	*Regions*	*Abbr.*	*Anatomical*	*No.*	*Regions*	*Abbr.*	*Anatomical*
1, 2	Precentral gyrus	PreCG	Sensorimotor	47, 48	Lingual gyrus	LING	Occipital
3, 4	Superior frontal gyrus, dorsolateral	SFGdor	Frontal	49, 50	Superior occipital gyrus	SOG	Occipital
5, 6	Superior frontal gyrus, orbital part	ORBsup	Frontal	51, 52	Middle occipital gyrus	MOG	Occipital
7, 8	Middle frontal gyrus	MFG	Frontal	53, 54	Inferior occipital gyrus	IOG	Occipital
9, 10	Middle frontal gyrus, orbital part	ORBmid	Frontal	55, 56	Fusiform gyrus	FFG	Temporal
11, 12	Inferior frontal gyrus, opercular part	IFGoperc	Frontal	57, 58	Postcentral gyrus	PoCG	Sensorimotor
13, 14	Inferior frontal gyrus, triangular part	IFGtriang	Frontal	59, 60	Superior parietal gyrus	SPG	Parietal
15, 16	Inferior frontal gyrus, orbital part	ORBinf	Frontal	61, 62	Inferior parietal	IPL	Parietal
17, 18	Rolandic operculum	ROL	Frontal	63, 64	Supramarginal gyrus	SMG	Parietal
19, 20	Supplementary motor area	SMA	Sensorimotor	65, 66	Angular gyrus	ANG	Parietal
21, 22	Olfactory cortex	OLF	Frontal	67, 68	Precuneus	PCUN	Parietal
23, 24	Superior frontal gyrus, medial	SFGmed	Frontal	69, 70	Paracentral lobule	PCL	Parietal
25, 26	Superior frontal gyrus, medial orbital	ORBsupmed	Frontal	71, 72	Caudate nucleus	CAU	Subcortical
27, 28	Gyrus rectus	REC	Frontal	73, 74	Lenticular nucleus, putamen	PUT	Subcortical
29, 30	Insula	INS	Subcortical	75, 76	Lenticular nucleus, pallidum	PAL	Subcortical
31, 32	Anterior cingulate & paracingulate gyri	ACG	Frontal	77, 78	Thalamus	THA	Subcortical
33, 34	Median cingulate & paracingulate gyri	DCG	Frontal	79, 80	Heschl gyrus	HES	Temporal
35, 36	Posterior cingulate gyrus	PCG	Parietal	81, 82	Superior temporal gyrus	STG	Temporal
37, 38	Hippocampus	HIP	Temporal	83, 84	Temporal pole: superior temporal gyrus	TPOsup	Temporal
39, 40	Parahippocampal gyrus	PHG	Temporal	85, 86	Middle temporal gyrus	MTG	Temporal
41, 42	Amygdala	AMYG	Subcortical	87, 88	Temporal pole: middle temporal gyrus	TPOmid	Temporal
43, 44	Calcarine fissure & surrounding cortex	CAL	Occipital	89, 90	Inferior temporal gyrus	ITG	Temporal
45, 46	Cuneus	CUN	Occipital				

**Table 3:** Correlations between the functional connectivity links involving the thalamus and the symptom severity scores (PANSS) and illness duration

*Links*		*Positive scale*		*Negative scale*		*General scale*		*Illness duration*	
		*Correlation*	P *value*	*Correlation*	P *value*	*Correlation*	P *value*	*Correlation*	P *value*
PoCG.L	THA.R	0.0090	0.8596	**0.1089**	**0.0396**	0.0194	0.7138	**0.1144**	**0.0305**
PoCG.L	THA.L	0.0076	0.8854	**0.1482**	**0.0049** a	0.0682	0.1971	**0.1503**	**0.0043** a
PoCG.R	THA.L	0.0288	0.5652	**0.1177**	**0.0259**	0.0547	0.3016	**0.1129**	**0.0325**
PoCG.R	THA.R	0.0701	0.1720	**0.1054**	**0.0465**	0.0280	0.6001	**0.1128**	**0.0325**
MFG.R	THA.R	0.0264	0.6255	0.0379	0.4754	−0.0858	0.0985	−0.0736	0.1597
PreCG.R	THA.L	−0.0076	0.8877	0.0738	0.1637	−0.0107	0.8392	0.0851	0.1066
PreCG.R	THA.R	−0.0009	0.9901	0.0922	0.0819	−0.0226	0.6606	0.0608	0.2518
PCL.L	THA.L	0.0003	0.9917	0.0561	0.2897	0.0494	0.3494	**0.1042**	**0.0484**
PCL.L	THA.R	0.0238	0.6463	0.0713	0.1770	−0.0322	0.5262	0.0811	0.1243
FFG.R	THA.R	0.0205	0.6947	0.0695	0.1822	0.0386	0.4521	0.0252	0.6351
ROL.L	THA.L	0.0231	0.6522	**0.1532**	**0.0036** a	0.1617	0.0021	−0.0695	0.1834
PreCG.L	THA.R	0.0075	0.8845	0.0863	0.1031	0.0281	0.5960	0.0171	0.7455
SFGmed.R	THA.L	−0.0607	0.2392	**−0.1212**	**0.0208**	**−0.1365**	**0.0085**	−0.0192	0.7121
FFG.L	THA.R	0.0236	0.6500	0.0457	0.3814	0.0062	0.8788	0.0014	0.9786
MFG.L	THA.L	−0.0318	0.5177	−0.0280	0.5959	**−0.1284**	**0.0132**	−0.0921	0.0812
THA.R	MTG.L	0.0091	0.8641	**0.1344**	**0.0097**	**0.1058**	**0.0432**	0.0056	0.9159
SPG.L	THA.R	0.0659	0.2121	−0.0011	0.9828	−0.0019	0.9615	0.0905	0.0852
MFG.L	THA.R	0.0093	0.8849	−0.0042	0.9385	−0.0941	0.0726	−0.0912	0.0840
PreCG.L	THA.L	−0.0446	0.3975	**0.1288**	**0.0144**	0.0106	0.8426	0.0623	0.2388
THA.L	MTG.L	−0.0050	0.9228	**0.1646**	**0.0017** a	0.0723	0.1699	0.0267	0.6157
PHG.R	THA.R	−0.0261	0.6249	−0.0091	0.8626	−0.0269	0.6111	0.0011	0.9818
PCL.R	THA.R	0.0630	0.2256	0.0449	0.3967	−0.0279	0.5738	**0.1195**	**0.0234**
ROL.R	THA.R	0.0923	0.0622	**0.1687**	**0.0013** a	**0.1647**	**0.0016** a	−0.0501	0.3375
PCL.R	THA.L	0.0547	0.2863	0.0013	0.9814	0.0660	0.2115	0.0789	0.1290
SFGmed.L	THA.L	**−0.1312**	**0.0116**	−0.1019	0.0533	**−0.1390**	**0.0078**	−0.0345	0.5118
ROL.R	THA.L	0.0657	0.1918	**0.1609**	**0.0021** a	**0.1593**	**0.0024** a	−0.0668	0.2058
MFG.R	THA.L	0.1164	0.0229	−0.0309	0.5606	−0.0569	0.2773	−0.1098	0.0379
PAL.R	THA.R	−0.0147	0.7820	−0.0207	0.6914	0.0642	0.2262	−0.0343	0.5172
FFG.R	THA.L	−0.0555	0.2949	0.0982	0.0605	0.0301	0.5566	0.0013	0.9710
THA.L	HES.L	0.0758	0.1349	**0.1182**	**0.0251**	**0.1619**	**0.0020** a	−0.0032	0.9526
THA.L	THA.R	0.1325	0.0110	**−0.1139**	**0.0306**	0.0785	0.1255	−0.0844	0.1096
THA.L	STG.L	0.0625	0.2188	**0.1502**	**0.0043** a	**0.1776**	**0.0007** a	−0.0270	0.6140
SFGmed.R	THA.R	**−0.1460**	**0.0046** a	**−0.1083**	**0.0401**	**−0.1513**	**0.0035** a	−0.0130	0.8050

Abbreviations: FFG, fusiform gyrus; MFG, middle frontal gyrus; PCL, paracentral lobule; PHG, parahippocampal gyrus; PoCG, postcentral gyrus; PreCG, precental gyrus; ROL, rolandic operculum; SFG.med, superior frontal gyrus, medial; SPG, superior parietal gyrus; THA, thalamus.

a False discovery rate correction *P*<0.05.Correlations with nonthalamic links shown in Supplementary Table S4.Uncorrected *P*-values less than 0.05 are indicated in bold.

## References

[bib1] Lanczik M, Keil G. Carl Wernicke's localization theory and its significance for the development of scientific psychiatry. Hist Psychiatry 1991; 2: 171–180. 11613217 10.1177/0957154X9100200604

[bib2] Cutting JC, Shepherd M. The Clinical roots of the schizophrenia concept: translations of seminal European contributions on schizophrenia. Cambridge University Press Archive: 1987.

[bib3] Liddle P, Friston K, Frith C, Hirsch S, Jones T, Frackowiak R. Patterns of cerebral blood flow in schizophrenia. Br J Psychiatry 1992; 160: 179–186. 1540757 10.1192/bjp.160.2.179

[bib4] Pettersson-Yeo W, Allen P, Benetti S, McGuire P, Mechelli A. Dysconnectivity in schizophrenia: where are we now? Neurosci Biobehav Rev 2011; 35: 1110–1124. 21115039 10.1016/j.neubiorev.2010.11.004

[bib5] Ioannidis JP, Trikalinos TA, Khoury MJ. Implications of small effect sizes of individual genetic variants on the design and interpretation of genetic association studies of complex diseases. Am J Epidemiol 2006; 164: 609–614. 16893921 10.1093/aje/kwj259

[bib6] Bora E, Fornito A, Radua J, Walterfang M, Seal M, Wood SJ et al. Neuroanatomical abnormalities in schizophrenia: a multimodal voxelwise meta-analysis and meta-regression analysis. Schizophr Res 2011; 127: 46–57. 21300524 10.1016/j.schres.2010.12.020

[bib7] Glahn DC, Laird AR, Ellison-Wright I, Thelen SM, Robinson JL, Lancaster JL et al. Meta-analysis of gray matter anomalies in schizophrenia: application of anatomic likelihood estimation and network analysis. Biol Psychiatry 2008; 64: 774–781. 18486104 10.1016/j.biopsych.2008.03.031 PMC5441233

[bib8] Palaniyappan L, Liddle PF. Does the salience network play a cardinal role in psychosis? An emerging hypothesis of insular dysfunction. J Psychiatry Neurosci 2012; 37: 17. 21693094 10.1503/jpn.100176 PMC3244495

[bib9] McKeown MJ, Hansen LK, Sejnowsk TJ. Independent component analysis of functional MRI: what is signal and what is noise? Curr Opin Neurobiol 2003; 13: 620–629. 14630228 10.1016/j.conb.2003.09.012 PMC2925426

[bib10] Mannell MV, Franco AR, Calhoun VD, Cañive JM, Thoma RJ, Mayer AR. Resting state and task‐induced deactivation: A methodological comparison in patients with schizophrenia and healthy controls. Hum Brain Mapp 2010; 31: 424–437. 19777578 10.1002/hbm.20876 PMC2826505

[bib11] Joel SE, Caffo BS, van Zijl P, Pekar JJ. On the relationship between seed‐based and ICA‐based measures of functional connectivity. Magn Reson Med 2011; 66: 644–657. 21394769 10.1002/mrm.22818 PMC3130118

[bib12] Liptak T. On the combination of independent tests. Magyar Tud Akad Mat Kutato Int Kozl 1958; 3: 171–197.

[bib13] Majeti R, Becker MW, Tian Q, Lee TL, Yan X, Liu R et al. Dysregulated gene expression networks in human acute myelogenous leukemia stem cells. Proc Natl Acad Sci USA 2009; 106: 3396–3401. 19218430 10.1073/pnas.0900089106 PMC2642659

[bib14] Karg K, Burmeister M, Shedden K, Sen S. The serotonin transporter promoter variant (5-HTTLPR), stress, and depression meta-analysis revisited: evidence of genetic moderation. Arch Gen Psychiatry 2011; 68: 444–454. 21199959 10.1001/archgenpsychiatry.2010.189 PMC3740203

[bib15] Richards JB, Waterworth D, O'Rahilly S, Hivert MF, Loos RJ, Perry JR et al. A genome-wide association study reveals variants in ARL15 that influence adiponectin levels. PLoS Genet 2009; 5: e1000768. 20011104 10.1371/journal.pgen.1000768 PMC2781107

[bib16] Hwang D, Rust AG, Ramsey S, Smith JJ, Leslie DM, Weston AD et al. A data integration methodology for systems biology. Proc Natl Acad Sci USA 2005; 102: 17296–17301. 16301537 10.1073/pnas.0508647102 PMC1297682

[bib17] Kevin KY, Cheung C, Chua SE, McAlonan GM. Can Asperger syndrome be distinguished from autism? An anatomic likelihood meta-analysis of MRI studies. J Psychiatry Neurosci 2011; 36: 412. 21406158 10.1503/jpn.100138 PMC3201995

[bib18] Tzourio-Mazoyer N, Landeau B, Papathanassiou D, Crivello F, Etard O, Delcroix N et al. Automated anatomical labeling of activations in SPM using a macroscopic anatomical parcellation of the MNI MRI single-subject brain. Neuroimage 2002; 15: 273–289. 11771995 10.1006/nimg.2001.0978

[bib19] Wang K, Liang M, Wang L, Tian L, Zhang X, Li K et al. Altered functional connectivity in early Alzheimer's disease: a resting-state fMRI study. Hum Brain Mapp 2007; 28: 967–978. 17133390 10.1002/hbm.20324 PMC6871392

[bib20] Bai F, Liao W, Watson DR, Shi Y, Wang Y, Yue C et al. Abnormal whole-brain functional connection in amnestic mild cognitive impairment patients. Behav Brain Res 2011; 216: 666–672. 20851147 10.1016/j.bbr.2010.09.010

[bib21] Cochran WG. The combination of estimates from different experiments. Biometrics 1954; 10: 101–129.

[bib22] Hedges LV, Vevea JL. Fixed-and random-effects models in meta-analysis. Psychol Methods 1998; 3: 486.

[bib23] Chang C-C, Lin C-J. LIBSVM: a library for support vector machines. ACM Trans Intell Syst Technol 2011; 2: 27.

[bib24] Garrity A, Pearlson G, McKiernan K, Lloyd D, Kiehl K, Calhoun V. Aberrant “default mode” functional connectivity in schizophrenia. Am J Psychiatry 2007; 164: 450–457. 17329470 10.1176/ajp.2007.164.3.450

[bib25] Bluhm RL, Miller J, Lanius RA, Osuch EA, Boksman K, Neufeld RW et al. Spontaneous low-frequency fluctuations in the BOLD signal in schizophrenic patients: anomalies in the default network. Schizophr Bull 2007; 33: 1004–1012. 17556752 10.1093/schbul/sbm052 PMC2632312

[bib26] Broyd SJ, Demanuele C, Debener S, Helps SK, James CJ, Sonuga-Barke EJ. Default-mode brain dysfunction in mental disorders: a systematic review. Neurosci Biobehav Rev 2009; 33: 279–296. 18824195 10.1016/j.neubiorev.2008.09.002

[bib27] Kim DI, Manoach DS, Mathalon DH, Turner JA, Mannell M, Brown GG et al. Dysregulation of working memory and default‐mode networks in schizophrenia using independent component analysis, an fBIRN and MCIC study. Hum Brain Mapp 2009; 30: 3795–3811. 19434601 10.1002/hbm.20807 PMC3058491

[bib28] Zhou Y, Liang M, Tian L, Wang K, Hao Y, Liu H et al. Functional disintegration in paranoid schizophrenia using resting-state fMRI. Schizophr Res 2007; 97: 194–205. 17628434 10.1016/j.schres.2007.05.029

[bib29] Woodward ND, Karbasforoushan H, Heckers S. Thalamocortical dysconnectivity in schizophrenia. Am J Psychiatry 2012; 169: 1092–1099. 23032387 10.1176/appi.ajp.2012.12010056 PMC3810300

[bib30] Welsh RC, Chen AC, Taylor SF. Low-frequency BOLD fluctuations demonstrate altered thalamocortical connectivity in schizophrenia. Schizophr Bull 2010; 36: 713–722. 18990709 10.1093/schbul/sbn145 PMC2894601

[bib31] Andreasen NC, O'Leary DS, Cizadlo T, Arndt S, Rezai K, Ponto LL et al. Schizophrenia and cognitive dysmetria: a positron-emission tomography study of dysfunctional prefrontal-thalamic-cerebellar circuitry. Proc Natl Acad Sci USA 1996; 93: 9985–9990. 8790444 10.1073/pnas.93.18.9985 PMC38542

[bib32] Anticevic A, Cole MW, Repovs G, Murray JD, Brumbaugh MS, Winkler AM et al. Characterizing thalamo-cortical disturbances in schizophrenia and bipolar illness. Cereb Cortex 2013; 24: 3116–3130. 23825317 10.1093/cercor/bht165 PMC4224238

[bib33] Marenco S, Stein JL, Savostyanova AA, Sambataro F, Tan HY, Goldman AL et al. Investigation of anatomical thalamo-cortical connectivity and FMRI activation in schizophrenia. Neuropsychopharmacology 2011; 37: 499–507. 21956440 10.1038/npp.2011.215 PMC3242311

[bib34] Chun S, Westmoreland JJ, Bayazitov IT, Eddins D, Pani AK, Smeyne RJ et al. Specific disruption of thalamic inputs to the auditory cortex in schizophrenia models. Science 2014; 344: 1178–1182. 24904170 10.1126/science.1253895 PMC4349506

[bib35] Shergill SS, Samson G, Bays PM, Frith CD, Wolpert DM. Evidence for sensory prediction deficits in schizophrenia. Am J Psychiatry 2005; 162: 2384–2386. 16330607 10.1176/appi.ajp.162.12.2384

[bib36] Chambon V, Pacherie E, Barbalat G, Jacquet P, Franck N, Farrer C. Mentalizing under influence: abnormal dependence on prior expectations in patients with schizophrenia. Brain 2011; 134: 3725–3738. 10.1093/brain/awr306 22108577

[bib37] Guller Y, Ferrarelli F, Shackman AJ, Sarasso S, Peterson MJ, Langheim FJ et al. Probing thalamic integrity in schizophrenia using concurrent transcranial magnetic stimulation and functional magnetic resonance imaging. Arch Gen Psychiatry 2012; 69: 662–671. 22393203 10.1001/archgenpsychiatry.2012.23 PMC3411883

[bib38] Spalletta G, Piras F, Rubino IA, Caltagirone C, Fagioli S. Fronto-thalamic volumetry markers of somatic delusions and hallucinations in schizophrenia. Psychiatry Res 2013; 212: 54–64. 23158777 10.1016/j.pscychresns.2012.04.015

[bib39] Stanghellini G. Embodiment and schizophrenia. World Psychiatry 2009; 8: 56–59. 19293962 10.1002/j.2051-5545.2009.tb00212.x PMC2652898

[bib40] Gallese V, Lakoff G. The brain's concepts: the role of the sensory-motor system in conceptual knowledge. Cogn Neuropsychol 2005; 22: 455–479. 21038261 10.1080/02643290442000310

[bib41] Meteyard L, Cuadrado SR, Bahrami B, Vigliocco G. Coming of age: a review of embodiment and the neuroscience of semantics. Cortex 2012; 48: 788–804. 21163473 10.1016/j.cortex.2010.11.002

[bib42] Nejad AB, Ebdrup BH, Glenthøj BY, Siebner HR. Brain connectivity studies in schizophrenia: unravelling the effects of antipsychotics. Curr Neuropharmacol 2012; 10: 219. 23449679 10.2174/157015912803217305 PMC3468876

[bib43] Lieberman JA. Is schizophrenia a neurodegenerative disorder? A clinical and neurobiological perspective. Biol Psychiatry 1999; 46: 729–739. 10494440 10.1016/s0006-3223(99)00147-x

