# Addendum: Voxel-based, brain-wide association study of aberrant functional connectivity in schizophrenia implicates thalamocortical circuitry

**DOI:** 10.1038/s41537-018-0060-x

**Published:** 2018-09-17

**Authors:** Wei Cheng, Lena Palaniyappan, Mingli Li, Keith M Kendrick, Jie Zhang, Qiang Luo, Zening Liu, Rongjun Yu, Wei Deng, Qiang Wang, Xiaohong Ma, Wanjun Guo, Susan Francis, Peter Liddle, Andrew R Mayer, Gunter Schumann, Tao Li, Jianfeng Feng

**Affiliations:** 1 Centre for Computational Systems Biology, Fudan University, Shanghai, China; 2 Centre for Translational Neuroimaging, Division of Psychiatry and Applied Psychology, Institute of Mental Health, University of Nottingham, Nottingham, UK; 3 The Mental Health Center and the Psychiatric Laboratory, West China Hospital, Sichuan University, Chengdu, China; 4 Key Laboratory for Neuroinformation, Ministry of Education of China, School of Life Science and Technology, University of Electronic Science and Technology of China, Chengdu, China; 5 Institute of Mental Health, Second Xiangya Hospital, Central South University, Changsha, China; 6 School of Psychology and Center for Studies of Psychological Application, South China Normal University, Guangzhou, China; 7 Sir Peter Mansfield MR Centre, University of Nottingham, Nottingham, UK; 8 The Mind Research Network, Albuquerque, USA; 9 Medical Research Council—Social, Genetic and Developmental Psychiatry Centre, Institute of Psychiatry, King’s College London, De Crespigny Park, London, UK; 10 Department of Computer Science, University of Warwick, Coventry, UK; 11 School of Life Science and the Collaborative Innovation Center for Brain Science, Fudan University, Shanghai, China

**Addendum to**: *npj Schizophrenia* 10.1038/npjschz.2015.16, Published online 6 May 2015

In the original published version of the manuscript, no data availability statement was included. This addendum has been added to include the following data availability statement “Data obtained from the patients in the Taiwan cohort were used with agreement from National Taiwan University Hospital. Data acquisition was funded by grant NSC99-3112-B-002-030 and was collected at National Taiwan University Hospital around 2010. Data obtained from the patients in the Huaxi cohort were used with agreement from Sichuan University. Data acquisition was funded by National Nature Science Foundation of China (81130024), National Key Technology R & D Program of the Ministry of Science and Technology of China during the 12th Five-Year Plan (2012BAI01B06). Reasonable requests for access to the data should be directed to Tao Li, The Mental Health Center and the Psychiatric Laboratory, West China Hospital, Sichuan University. Data obtained from the patients in the Xiangya cohort were used with agreement from the Second Xiangya Hospital of Central South University in China. Reasonable requests for access to the data should be directed to Zening Liu, Institute of Mental Health, Second Xiangya Hospital, Central South University. Data obtained from the patients in the Nottingham cohort were used with agreement from Nottingham University. Data acquisition was funded by MRC (grant reference G0601442). Reasonable requests for access to the data should be directed to Peter F Liddle, Division of Psychiatry and Applied Psychology, University of Nottingham.”

