# Supplementary material for: Voxel-based, brain-wide association study of aberrant functional connectivity in schizophrenia implicates thalamocortical circuitry
Source: NPJ Schizophr. 2015 May 6;1:15016–. doi: 10.1038/npjschz.2015.16 (PMC4849447; doi:10.1038/npjschz.2015.16)
Supplement: Supplementary Information [file npjschz201516-s1.doc]

Supplementary Information:

**Voxel-based, Brain-wide Association Study of Aberrant Functional Connectivity in Schizophrenia Implicates Thalamocortical Circuitry**

Wei Cheng, Ph.D ^1, #^, Lena Palaniyappan, MRCPsych, Ph.D ^2, #^, Mingli Li, Ph.D ^3, #^, Keith M. Kendrick, Jie Zhang ^1^, Qiang Luo ^1^, Ph.D ^6, #^, Zening Liu, Ph.D ^9^, Rongjun Yu, Ph.D ^10^, Wei Deng, Ph.D ^3^, Qiang Wang, Ph.D ^3^, Xiaohong Ma, Ph.D ^3^, Wanjun Guo, Ph.D ^3^, Susan Francis, Ph.D ^7^, Peter Liddle, Ph.D ^2^, Andrew R. Mayer, Ph.D ^8^, Gunter Schumann, Ph.D ^4^, Tao Li, Ph.D ^3, *^, Jianfeng Feng, Ph.D ^1, 5,11,*^

**Method**

**Subjects**

Functional scans collected under resting-state conditions from 415 patients with chronic schizophrenia and 405 healthy control subjects were included in the study. After quality control, a total of 773 subjects was included in following analysis (375 healthy controls and 398 schizophrenia patients). The dataset is comprised of patient and healthy control data from five sources. The first dataset was collected at Xiangya hospital in China (83 schizophrenia patients and 60 healthy controls); the second dataset from the National Taiwan University Hospital in Taiwan (69 patients and 62 healthy controls); the third dataset was from the University of Nottingham in the UK (32 patients and 36 healthy controls) with data collected by the Early Intervention in Psychosis Team and community based Mental healthcare Teams in Nottinghamshire. The fourth dataset was also collected at Huaxi hospital in China (178 schizophrenia patients and 180 healthy controls). The last one is from The Center for Biomedical Research Excellence (COBRE), which a public shared MRI data of schizophrenia. All patients were identified according to the DSM-IV diagnostic criteria by qualified psychiatrists using a best estimate procedure that utilized all available clinical information including a diagnostic interview, clinical case notes, treating clinician’s observations and informant reports. Symptom severity was measured using the Positive and Negative Syndrome Scale (PANSS) assessment (Xiangya, Huaxi, Taiwan and COBRE) or Signs and Symptoms of Psychotic Illness (SSPI) (Nottingham) given to all patients either one week before the MRI scan or one week after it. However, 31 patients did not have a PANSS assessment completed. All healthy controls were assessed in accordance with DSM-IV criteria as being free of schizophrenia and other Axis I disorders and none had any neurological diseases, suffered from clinically significant head trauma or had a history of substance dependence. Written informed consent was obtained from all individual participants, and research procedures and ethical guidelines were followed in accordance with the Institutional Review Boards (IRB) of the respective hospitals (Taiwan, China (mainland)), USA and the National Research Ethics Committee (UK). All participants provided written informed consent prior to entering the study. Details of the acquisition of these datasets are provided below.

**Taiwan**

Schizophrenia data was collected by the National Taiwan University Hospital in China, and included 69 patients with chronic schizophrenia and 62 healthy control subjects. Symptom severity was measured using the Positive and Negative Syndrome Scale (PANSS) administered either one week before or after the MRI scan. Symptom severity was measured using the Positive and Negative Syndrome Scale (PANSS) assessment which was given to all schizophrenic participants either one week before or one week after their MRI scan. However, five patients were not able to complete their PANSS assessment because of their poor health. All patients were taking antipsychotic medication.

Sixty-two (25 males and 37 females) healthy control subjects were also recruited. All of the controls were assessed in accordance with DSM-IV criteria as being free of schizophrenia and other Axis I disorders. None had any neurological diseases or suffered from clinically significant head trauma, and none had a history of any substance dependence. Written informed consent was obtained from all individual participants, and research procedures and ethical guidelines were followed in accordance with the Institutional Review Board (IRB) of the National Taiwan University hospital.

**Xiangya**

Schizophrenia data was collected by Second Xiangya Hospital of Central South University in China. All patients were identified according to the DSM-IV diagnostic criteria by qualified psychiatrists. Symptom severity was measured using the Positive and Negative Syndrome Scale (PANSS) administered either one week before or after the MRI scan. A total of 26 patients had no PANSS records due to incomplete records or because they did not have a PANSS assessment carried out. 22 patients were treatment naive, and 61 were on antipsychotic medication.

60 healthy control subjects were also recruited. All controls were assessed in accordance with DSM-IV criteria as being free of schizophrenia and other Axis I disorders. None had any neurological diseases or suffered from clinically significant head trauma or had a history of any substance dependence. Written informed consent was obtained from all individual participants, and research procedures and ethical guidelines were followed in accordance with the Institutional Review Board (IRB) of the hospital.

**Nottingham**

Patients with psychotic disorders were referred by the Early Intervention in Psychosis Team and other community based Mental Healthcare Teams in Nottinghamshire and Leicestershire, England. The clinical features were confirmed through retrospective case note review, standardized symptom assessment using Signs and Symptoms in Psychotic Illness scale (SSPI), and several clinical consensus meetings among two or four research psychiatrists trained in use of research diagnostic criteria. To allow a comparison with the other two datasets a positive, negative and general symptom scores were calculated using individual items from the SSPI which overlapped with individual PANSS items (Positive scale include: 1. Elated Mood, 2. Overactivity, 3. Pressure of Speech, 4. Insomnia, 5. Hostility, 6. Peculiar Behaviour, 7. Delusions, 8. Hallucinations. Negative scale include: 9. Underactivity, 10. Blunted Affect, 11. Poverty of Speech, 12. Anhedonia. General Psychopathology scale include: 13. Anxiety, 14. Depression, 15. Somatic Complaints.). Diagnoses were assigned according to DSMIV criteria, at a consensus meeting using the Best Diagnostic Estimate procedure. The study was conducted with ethical approval by the National Research Ethics Committee, Derbyshire, UK. Written informed consent was obtained from all study subjects in accord with the procedure approved by the Ethics Committee.

**Huaxi**

In total 358 subjects including 178 schizophrenia patients (90 males, 88 females) and 180 healthy controls (95 males, 85 females) participated in this study. All patients were recruited at the Mental Health Center of West China Hospital, Sichuan University from August 2005 to October 2012. The patients were interviewed by an experienced psychiatrist using the Structured Clinical Interview for DSM-IV. Patients diagnosed with schizophreniform psychosis were followed up for at least 6 months and all met the DSM-IV criteria for schizophrenia. Psychopathology associated with schizophrenia was evaluated using the PANSS. 2. Most of patients in this sample were antipsychotic naïve, and small number (<5%) were prescribed antipsychotics at a low dose (25–75 mg daily dose equivalents of chlorpromazine) for <5 days before MRI scanning.

Healthy controls were recruited from the local area through poster advertisement. All controls were screened with the SCID non-patient version for the lifetime absence of psychiatric illnesses. Subjects with organic brain disorders, alcohol and/or drug abuse, pregnancy or any other severe physical illness such as brain tumor or epilepsy were excluded from the study. The study was approved by the ethical committee of the West China Hospital of Sichuan University. All patients and controls provided written informed consent.

**COBRE**

[The Center for Biomedical Research Excellence (COBRE)](http://cobre.mrn.org/) is contributing raw anatomical and functional MR data from 53 patients with Schizophrenia and 67 healthy controls (ages ranging from 18 to 65 in each group). All subjects were screened and excluded if they had: history of neurological disorder, history of mental retardation, history of severe head trauma with more than 5 minutes loss of consciousness, history of substance abuse or dependence within the last 12 months. Diagnostic information was collected using the Structured Clinical Interview used for DSM Disorders (SCID).

**Data Acquisition**

**Taiwan**

All data were collected on a 3 Tesla Siemens Trio Tim scanner with an eight channel phase array head coil. Axial resting state fMRI data were acquired using a gradient echo echo-planar imaging (GE-EPI) sequence. The following parameters were used: repetition time (TR)/ echo time (TE) = 2000 ms/30 ms, flip angle (FA) = 90º, matrix = 64 × 64, FOV = 24 × 24 cm2, slice thickness = 4 mm, and slice gap = 0.4 mm. A total of 30 slices were used to cover the whole brain. Each scan contained 250 volumes. Subjects were instructed to relax, hold still, keep their eyes closed without falling asleep, and think of nothing in particular.

**Xiangya**

Image data were acquired on a 1.5 Tesla Siemens MRI scanner. A total of 180 volumes of axial GE-EPI images were obtained (TR/TE = 2000 ms/40 ms, FA = 90°, matrix = 64 × 64, FOV = 24 × 24cm2, 20 slices 20 of slice thickness = 5mm, and slice gap= 1 mm). Each scan contained 180 volumes.

**Nottingham**

Data was acquired on a 3 Tesla Philips Achieva MRI scanner (Philips, Netherlands) using an eight-channel SENSE head coil. To enhance sensitivity, dual-echo GE-EPI were acquired (TR = 2500 ms with TE1/TE2 = 20/48 ms, FA = 85°, SENSE factor 2 in anterior-posterior direction, matrix = 85 × 85, FOV = 25.5 × 25.5 cm2, with an in-plane resolution of 3 mm × 3 mm and, 40 contiguous axial slices acquired in descending order with slice thickness of 4 mm. Each scan comprised 410 volumes.

**Huaxi**

All participants were scanned using a 3-T MRI system (EXCITE, General Electric, USA). Functional MRI (fMRI) images were obtained using a gradient–echo echo-planar imaging (EPI) sequence [repetition time (TR)/echo time (TE)=2000/30 ms, flip angle=90x, slice thickness=5 mm, no slice gap, in-plane resolution=3.75r3.75 mm2, matrix size=64r64, field of view (FOV)=240r240 mm2, slice number=30]. Each resting-state fMRI scan contained 200 image volumes.

**COBRE**

Rest data was collected with single-shot full k-space echo-planar imaging (EPI) with ramp sampling correction using the intercomissural line (AC-PC) as a reference (TR: 2 s, TE: 29 ms, matrix size: 64x64, 32 slices, voxel size: 3 × 3 × 4 mm3). A multi-echo MPRAGE (MEMPR) sequence was used with the following parameters: TR/TE/TI = 2530/[1.64, 3.5, 5.36, 7.22, 9.08]/900 ms, flip angle = 7°, FOV = 256x256 mm, Slab thickness = 176 mm, Matrix = 256x256x176, Voxel size =1 × 1 × 1 mm, Number of echos = 5, Pixel bandwidth =650 Hz, Total scan time = 6 min. With 5 echoes, the TR, TI and time to encode partitions for the MEMPR are similar to that of a conventional MPRAGE, resulting in similar GM/WM/CSF contrast.

**Data Preprocessing**

Preprocessing and statistical analysis of functional images were carried out using the Statistical Parametric Mapping package (SPM8, Wellcome Department for Imaging Neuroscience, London, UK). For each individual participant’s dataset, the first 10 image volumes were discarded to allow the fMRI signal to reach a steady state. Initial analysis included slice time correction and Motion realignment. The resulting images were then spatially normalized to the Montreal Neurological Institute (MNI) EPI template in SPM8, resampled to 3×3×3 *mm*^3^, and subsequently smoothed with an isotropic Gaussian kernel (FWHM 8 mm).

To remove the sources of spurious correlations present in resting- state BOLD data, all fMRI time-series underwent high-pass temporal filtering (0.01 Hz), nuisance signal removal from ventricles, deep white matter, global mean signal (GMS) and 6 rigid-body motion correction parameters, followed by low-pass temporal filtering (0.08 Hz). In addition, given the growing concerns that excessive moment can impact between-group differences, we use four procedure to achieve motion correction. In the first step, we carry out three-dimensional motion correction by aligning each functional volume to the mean image of all volumes and any data affected by head motion of > 3 mm or rotation of > $3^{\text{o}}$ was excluded. In the second step, we implemented additional careful volume censoring (“scrubbing”) movement correction as reported by Power et al. ^1^ to ensure that head-motion artifacts are not driving observed effects[^1^](#_ENREF_1). The mean framewise displacement (FD) was computed with FD threshold for displacement being 0.5. In addition to the frame corresponding to the displaced time point, 1 preceding and 2 succeeding time points were also deleted to reduce the ‘spill-over’ effect of head movements. Thirdly, subjects with >10% displaced frames flagged were completely excluded from the analysis as it is likely that such high-level of movement would have had an influence on several volumes. Finally, the mean displacements after scrubbing were computed as root-mean-square of the translation parameters and rotation parameters (computed as the average of the absolute value of the Euler angle of the rotation of each brain volume as compared to the previous volume)[^2^](#_ENREF_2). The total root mean square displacements compared between the 2 groups in the overall sample did not show significant differences (Table S8). Despite this, it is possible that the remaining differences in the motion parameters can influence the connectivity measures within individual datasets. As a result, we used the root mean square displacement as a covariate when comparing the 2 groups during statistical analysis.

**The global signal**

At present, there is no consensus in the field with regard to the removal of global signal when computing connectivity metrics. Global signal removal can increase the frequency of pairwise negative correlation coefficients across the brain when instantaneous relationships are computed[^3^](#_ENREF_3); nevertheless, it has been shown to reduce physiological noise from resting fMRI, thus improving its reliability[^4-6^](#_ENREF_4). In particular, removal of global signal also reduces the variance due to movement related effects[^6^](#_ENREF_6). The major argument against the removal of global signal is the introduction of spurious correlations; for the current study where the primary effect of interest is the difference between 2 groups in the magnitude of the connectivity strength (irrespective of the sign of the coefficients), it is essential that both groups are treated identically in terms of global signal removal to avoid this bias. We regressed out the global signal in both groups using identical approach, thus reducing the possibility of introducing spurious group differences. Furthermore, in line with Murphy et al.[^7^](#_ENREF_7), we have refrained from interpreting negative correlation coefficients as representative of anticorrelations; instead we consider them only on relative terms, as values representing coefficients of lower magnitude compared to the positive correlations.

**Table S1.** Summary of the published resting-state connectivity literature in schizophrenia to date (table is updated from Pettersson-Yeo et al. 2011).[^8^](#_ENREF_8) In order to identify suitable publications, an online search of the Pubmed, using the search terms ‘schizophrenia’, ‘resting state fMRI’ and ‘functional connectivity’. 35 studies have been published targeting changes in functional connectivity of schizophrenia using resting state fMRI data. The majority of schizophrenia studies included in this study report reduced connectivity especially the functional connectivities between frontal and other brain regions in patients relative to health controls. However, the conclusions drawn from most of these studies were based on seed-based analysis or independent component analysis, and the statistical power was not very strong due to the relatively small sample size in individual site-specific studies. Furthermore, within and amongst the results a range of inconsistencies in findings about the altered pattern of connectivity and the localization of the brain areas involved are evident.

| **No.** | **Author** | **Sample size** | | **Method** | **Functional Connectivity** | | **Statistic** |
| --- | --- | --- | --- | --- | --- | --- | --- |
|  |  | **HC** | **SZ** |  |  |  |  |
| **1** | **Our study** | **405** | **415** | **whole brain voxel-wise functional connectivity analysis** | **Thalamus** --- **Motor and sensory cortex** |  | **p = 10^-18^** |
|  |  |  |  |  | **Thalamus** --- **Frontal gyrus** |  |  |
| **2** | Anticevic et al., 2014 | 90 | 90 | ROI-wise correlation analysis | Mediodorsal and lateral geniculate nucleus thalamic nuclei --- Sensorymotor regions |  | Max z = 6 |
|  |  |  |  |  | Mediodorsal and lateral geniculate nucleus thalamic nuclei --- Prefrontalstriatal and cerebellar clusters |  |  |
| **3** | Manoliu et al., 2014 | 20 | 18 | Independent component analysis | Default mode network --- Central executive network,  within the default mode network |  | p < 0.001  Max t = 5 |
|  |  |  |  |  | Salience network --- Default mode and central executive network |  |  |
| **4** | Pu et al., 2014 | 60 | 83 | Parcellated region correlation | Posterior cingulate gyrus --- Precuneus,  Left amygdala --- Right amygdala |  | Min p = 4.2 × 10^−4^ |
| **5** | Guo et al., 2014 | 50 | 49 | Granger causality analysis | Medial prefrontal cortex --- Middle temporal gyrus and angular gyrus; anterior cingulate cortex --- Supramarginal gyrus, superior occipital gyrus, supramarginal gyrus |  | Max t = 4.8 |
|  |  |  |  |  | Anterior cingulate cortex --- Cerebellum |  |  |
| **6** | Alonso-Solís et al., 2014 | 20 | 33 | ROI-wise correlation analysis | Posterior inferior parietal lobule --- Occipital fusiform gyrus, lingual gyrus and occipital pole  Retrosplenial cortex --- Lateral occipital cortex, intracalcarine cortex, occipital fusiform gyrus and lingual gyrus |  | z > 2.3; |
| **7** | Chang et al., 2014 | 22 | 24 | Independent component analysis | Right-lateralized portions of fronto-parietal network --- Anterior portions of default mode network |  | p = 0.027 |
| **8** | Wang et al., 2014 | 60 | 60 | ROI-wise correlation analysis | Cerebellar --- Thalamus, middle frontal gyri, anterior  cingulate cortex and supplementary motor area |  | Max t = 6.2 |
| **9** | Liu et al., 2014 | 18 | 18 | ROI-wise correlation analysis | Amygdala --- Rostral prefrontal cortex |  | Max t = 5.34 |
|  |  |  |  |  | Amygdala --- Dorsal lateral prefrontal cortex and middle cingulate cortex |  |  |
| **10** | Klingner et al., 2014 | 22 | 22 | Parcellated region correlation | Thalamus --- Ventrolateral prefrontal, secondary motor and sensory cortical areas |  | Max t = 5.2 |
| **11** | Moran et al., 2013 | 44 | 44 | ROI-wise correlation analysis | Insula --- Default mode network (including mPFC, PCC, and lateral parietal cortex) |  | Max t = 4.8 |
| **12** | Mwansisya et al., 2013 | 33 | 41 | Parcellated region correlation | Interhemispheric connection in the globus pallidus, medial frontal gyrus and inferior temporal gyrus |  | p < 0.05 |
| **13** | Orliac et al., 2013 | 26 | 26 | Independent component analyses | Paracingulate cortex (default mode network)  Putamen and pallidum (salience network) |  | Max t = 5.5 |
| **14** | Su et al., 2013 | 25 | 25 | ROI-wise correlation analysis | Dorsolateral prefrontal cortex --- Caudate nucleus, middle frontal gyrus, precentral gyrus, cerebellum and inferior frontal gyrus |  | p < 0.05 |
| **15** | Oertel-Knöchel et al., 2013 | 24 | 24 | ROI-wise correlation analysis | Planum temporale --- Superior temporal gyrus, inferior frontal gyrus, precentral gyrus, cingulate gyrus and thalamus |  | p < 0.05 |
| **16** | Chen et al., 2013 | 36 | 36 | ROI-wise correlation analysis | Cerebellar --- Thalamus |  | p < 0.001 |
| **17** | Tang et al., 2013 | 32 | 32 | Independent component analyses | Medial frontal gyrus --- Other areas of the default mode network |  | Max t = 4.38 |
| **18** | Liemburg et al., 2012 | 30 | 45 | Independent component analysis | Anterior cingulate --- Broca's networks |  | Min p = 0.0005 |
|  |  |  |  |  | Superior temporal gyrus --- Broca's networks |  |  |
| **19** | Tu et al., 2012 | 30 | 30 | ROI wise correlation analysis | Putamen --- Dorsal anterior cingulate, prefrontal cortex, insula, and inferior parietal lobule |  | p < 0.014 |
| **20** | Liu et al., 2012 | 25 | 25 | ROI wise correlation analysis | inferior temporal gyri --- posterior cingulated cortex and precuneus; lateral parietal cortex--- medial prefrontal cortex  Left inferior temporal gyrus --- right inferior temporal gyrus |  | p < 0.05 |
| **21** | Yan et al., 2012 | 30 | 33 | ROI wise correlation analysis | cognitive division of anterior cingulate cortex --- putamen, caudate, thalamus, anterior cingulate and medial prefrontal cortex |  | Max t = 6.3 |
|  |  |  |  |  | cognitive division of anterior cingulate cortex --- precuneus and sensorimotor cortex |  |  |
| **22** | Woodward et al., 2012 | 77 | 62 | ROI wise correlation analysis | Thalamus --- Motor and somatosensory cortex |  | Min p = 10^-6^ |
|  |  |  |  |  | Thalamus --- Prefrontal cortex |  |  |
| **23** | Hu Liu et al., 2011 | 18 | 18 | ROI wise correlation analysis | Laterobasal and superficial amygdala --- Rostral prefrontal cortex |  | Max t = 5.5 |
|  |  |  |  |  | Amygdala --- Dorsolateral prefrontal cortex and middle cingulate cortex |  |  |
| **24** | Cole et al., 2011 | 22 | 23 | ROI-wise correlation analysis | Dorsolateral prefrontal cortex --- Sensory, semantic, motor regions, cerebellum, and mid temporal cortex |  | Max t = 2.7 |
|  |  |  |  |  | Within prefrontal cortex connectivity |  |  |
| **25** | Collin et al., 2011 | 41 | 62 | ROI-wise correlation analysis | Cerebellum --- Lingual gyrus |  | p < 0.0025 |
|  |  |  |  |  | Cerebellum --- Inferior frontal gyrus, supplementary motor area, middle cingulate gyrus, hippocampus and thalamus |  |  |
| **26** | Liu et al., 2011 | 10 | 10 | ROI-wise correlation analysis | Cerebellum --- Middle temporal gyrus, cerebellum, middle cingulate cortex, paracentral lobule and thalamus |  | Max t = 11.45 |
| **27** | Hoptman et al., 2010 | 21 | 25 | ROI wise correlation analysis | Amygdala --- Anterior cingulate gyrus, inferior frontal gyrus, medial frontal gyrus, middle frontal gyrus and lentiform nuclei |  | Max z = 4.21 |
| **28** | Öngür et al., 2010 | 15 | 14 | Independent component analysis | Default mode network --- Frontal Polar cortex, dorsolateral prefrontal cortex and basal ganglia |  | p < 0.001  Max z = 4.57 |
|  |  |  |  |  | Default mode network --- Dorsal anterior cingulate |  |  |
| **29** | Rotarska-Jagiela et al., 2010 | 16 | 16 | Independent component analysis | Parietal lobe (within) |  | Min p = 0.0027 |
|  |  |  |  |  | Posterior cingulate gyrus, hippocampus, middle frontal gyrus (within), Frontal lobe --- Parietal lobe |  |  |
| **30** | Vercammen et al., 2010 | 27 | 27 | ROI wise correlation analysis | Temporo-parietal junction --- Homotope of Broca’s area |  | p = 0.004 |
| **31** | Camchong et al., 2009 | 29 | 29 | Independent component analysis | Medial frontal gyrus --- Default mode network  Anterior cingulate gyrus --- Default mode network |  | p < 0.05 |
| **32** | Gavrilescu et al., 2009 | 14 | 12 | ROI wise correlation analysis | Right primary auditory cortices --- Left primary auditory cortices  Right secondary auditory cortices --- Left secondary auditory cortices |  | p < 0.0001 |
| **33** | Bluhm et al., 2009 | 17 | 17 | ROI wise correlation analysis | Retrosplenium --- Precuneus, cingulate gyrus, superior temporal gyrus, lingual gyrus, cerebellum, superior parietal lobule and medial frontal gyrus |  | Max z = 3.97 |
| **34** | Zhou et al., 2008 | 14 | 17 | ROI wise correlation analysis | Hippocampus --- Posterior cingulate cortex, superior temporal gyrus, parahippocampus, medial temporal pole, middle occipital gyrus, medial prefrontal cortex and cerebellum |  | Max t = 5.61 |
| **35** | Zhou et al., 2007 | 17 | 17 | ROI wise correlation analysis | Dorsolateral prefrontal cortex --- Mid-posterior temporal lobe and the paralimbic regions |  | Max t = 6 |
|  |  |  |  |  | Dorsolateral prefrontal cortex --- Parietal lobe, posterior cingulate cortex, thalamus and striatum |  |  |

**Table S2. The names, abbreviations and anatomical classification of the regions of interest (ROIs).**

| **NO.** | **Regions** | **Abbr.** | **Anatomical** | **NO.** | **Regions** | **Abbr.** | **Anatomical** |
| --- | --- | --- | --- | --- | --- | --- | --- |
| 1, 2 | Precental gyrus | PreCG | Sensorimotor | 47, 48 | Lingual gyrus | LING | Occipital |
| 3, 4 | Superior frontal gyrus, dorsolateral | SFGdor | Fontal | 49, 50 | Superior occipital gyrus | SOG | Occipital |
| 5, 6 | Superior frontal gyrus, orbital part | ORBsup | Fontal | 51, 52 | Middle occipital gyrus | MOG | Occipital |
| 7, 8 | Middle frontal gyrus | MFG | Fontal | 53, 54 | Inferior occipital gyrus | IOG | Occipital |
| 9, 10 | Middle frontal gyrus, orbital part | ORBmid | Fontal | 55, 56 | Fusiform gyrus | FFG | Temporal |
| 11, 12 | Inferior frontal gyrus, opercular part | IFGoperc | Fontal | 57, 58 | Postcentral gyrus | PoCG | Sensorimotor |
| 13, 14 | Inferior frontal gyrus, triangular part | IFGtriang | Fontal | 59, 60 | Superior parietal gyrus | SPG | Parietal |
| 15, 16 | Inferior frontal gyrus, orbital part | ORBinf | Fontal | 61, 62 | Inferior parietal | IPL | Parietal |
| 17, 18 | Rolandic operculum | ROL | Fontal | 63, 64 | Supramarginal gyrus | SMG | Parietal |
| 19, 20 | Supplementary motor area | SMA | Sensorimotor | 65, 66 | Angular gyrus | ANG | Parietal |
| 21, 22 | Olfactory cortex | OLF | Fontal | 67, 68 | Precuneus | PCUN | Parietal |
| 23, 24 | Superior frontal gyrus, medial | SFGmed | Fontal | 69, 70 | Paracentral lobule | PCL | Parietal |
| 25, 26 | Superior frontal gyrus, medial orbital | ORBsupmed | Fontal | 71, 72 | Caudate nucleus | CAU | Subcortical |
| 27, 28 | Gyrus rectus | REC | Fontal | 73, 74 | Lenticular nucleus, putamen | PUT | Subcortical |
| 29, 30 | Insula | INS | Subcortical | 75, 76 | Lenticular nucleus, pallidum | PAL | Subcortical |
| 31, 32 | Anterior cingulate & paracingulate gyri | ACG | Fontal | 77, 78 | Thalamus | THA | Subcortical |
| 33, 34 | Median cingulate & paracingulate gyri | DCG | Fontal | 79, 80 | Heschl gyrus | HES | Temporal |
| 35, 36 | Posterior cingulate gyrus | PCG | Parietal | 81, 82 | Superior temporal gyrus | STG | Temporal |
| 37, 38 | Hippocampus | HIP | Temporal | 83, 84 | Temporal pole: superior temporal gyrus | TPOsup | Temporal |
| 39, 40 | Parahippocampal gyrus | PHG | Temporal | 85, 86 | Middle temporal gyrus | MTG | Temporal |
| 41, 42 | Amygdala | AMYG | Subcortical | 87, 88 | Temporal pole: middle temporal gyrus | TPOmid | Temporal |
| 43, 44 | Calcarine fissure & surrounding cortex | CAL | Occipital | 89, 90 | Inferior temporal gyrus | ITG | Temporal |
| 45, 46 | Cuneus | CUN | Occipital |  |  |  | |

**Table S3.** Significant regions in the voxel-based whole brain analysis.

| **No.** | **Areas** | **Cluster size**  **#Voxels** | **Peak MA value** | **MNI coordinates (Peak)** |
| --- | --- | --- | --- | --- |
| **Cluster 1** | Pallidum_L, Putamen_L | 23 | 60 | -12 3 3 |
| **Cluster 2** | Frontal_Inf_Orb_L, Frontal_Inf_Tri_L, Frontal_Mid_Orb_L | 33 | 50 | -42 39 -3 |
| **Cluster 3** | Thalamus_R, Thalamus_L, Caudate_R, Pallidum_R, Putamen_R, | 442 | 222 | -12 -18 6 |
| **Cluster 4** | Frontal_Sup_Medial_R, Frontal_Sup_Medial_L, Cingulum_Ant_L, Cingulum_Ant_R | 122 | 22 | 6 51 18 |
| **Cluster 5** | Caudate_L | 20 | 49 | -15 0 18 |
| **Cluster 6** | Postcentral_R, Precentral_R, Parietal_Inf_R, Rolandic_Oper_R, SupraMarginal_R | 297 | 40 | 57 -9 27 |
| **Cluster 7** | Precuneus_R, Cingulum_Post_R, Cingulum_Mid_R | 67 | 21 | 6 -54 30 |
| **Cluster 8** | Postcentral_L, Parietal_Inf_L, Precentral_L, SupraMarginal_L | 167 | 61 | -48 -27 42 |
| **Cluster 9** | Frontal_Inf_Tri_R, Frontal_Mid_R | 50 | 68 | 54 24 27 |
| **Cluster 10** | Paracentral_Lobule_R, Postcentral_R, Supp_Motor_Area_R, Precentral_R | 43 | 19 | 6 -21 69 |
| **Cluster 11** | Postcentral_L | 20 | 14 | -36 -36 69 |

**Table S4.** Results of AAL-based meta-analysis.

| **Links** | | **P value of FE*** | **P value of RE*** | **Z value** | **P value of Hete.*** | **P value of sites** | | | | |
| --- | --- | --- | --- | --- | --- | --- | --- | --- | --- | --- |
|  |  |  |  |  |  | **Huaxi** | **COBRE** | **Taiwan** | **Xiangya** | **Nottingham** |
| PoCG.L | THA.R | 3.01E-15 | 7.73E-05 | 7.89 | 2.36E-04 | 2.51E-02 | 1.33E-06 | 5.01E-09 | 2.99E-03 | 2.99E-03 |
| PoCG.L | THA.L | 1.70E-14 | 5.90E-05 | 7.67 | 6.69E-04 | 1.88E-02 | 2.67E-05 | 7.53E-09 | 2.87E-03 | 3.49E-03 |
| PoCG.R | THA.L | 4.02E-14 | 1.23E-04 | 7.56 | 3.09E-04 | 3.33E-02 | 4.65E-06 | 2.88E-08 | 2.53E-03 | 5.53E-03 |
| PoCG.R | THA.R | 2.91E-13 | 2.31E-04 | 7.30 | 1.62E-04 | 8.10E-02 | 1.95E-06 | 6.80E-08 | 6.09E-03 | 2.03E-03 |
| IFGoperc.R | TPOmid.R | 1.36E-10 | 1.33E-04 | 6.42 | 5.79E-02 | 1.46E-07 | 2.68E-02 | 5.75E-04 | 1.99E-01 | 4.13E-01 |
| MFG.R | THA.R | 3.89E-10 | 1.43E-02 | -6.26 | 3.91E-09 | 8.41E-02 | 9.32E-07 | 9.90E-10 | 8.59E-01 | 7.70E-03 |
| PreCG.R | THA.L | 1.19E-09 | 7.63E-04 | 6.08 | 2.72E-03 | 9.95E-02 | 9.96E-04 | 1.50E-06 | 1.34E-03 | 2.01E-01 |
| PreCG.R | THA.R | 2.21E-09 | 1.73E-03 | 5.98 | 5.25E-04 | 2.46E-01 | 5.69E-04 | 4.28E-07 | 1.68E-03 | 1.24E-01 |
| PCL.L | THA.L | 2.44E-09 | 6.94E-05 | 5.97 | 2.47E-02 | 2.30E-02 | 1.15E-04 | 3.33E-04 | 2.14E-01 | 8.17E-04 |
| IFGtriang.R | TPOmid.R | 3.95E-09 | 3.69E-07 | 5.89 | 3.08E-01 | 1.23E-05 | 1.43E-02 | 1.33E-02 | 1.54E-02 | 7.70E-01 |
| PCL.L | THA.R | 2.45E-08 | 3.10E-04 | 5.58 | 1.46E-02 | 2.93E-02 | 1.95E-04 | 1.25E-03 | 5.54E-01 | 1.15E-04 |
| INS.L | PUT.R | 2.62E-08 | 6.65E-06 | -5.56 | 6.52E-01 | 6.26E-04 | 6.84E-02 | 8.60E-03 | 4.19E-03 | 2.19E-01 |
| ORBinf.R | INS.R | 2.70E-08 | 8.92E-07 | -5.56 | 5.92E-01 | 1.20E-02 | 2.33E-02 | 2.12E-02 | 6.88E-04 | 2.51E-02 |
| ORBinf.R | ROL.L | 3.10E-08 | 8.91E-07 | -5.54 | 5.54E-01 | 5.12E-04 | 2.02E-01 | 9.44E-02 | 7.07E-05 | 1.46E-01 |
| ORBinf.R | INS.L | 3.95E-08 | 3.91E-07 | -5.49 | 3.12E-01 | 2.70E-02 | 1.21E-03 | 3.72E-02 | 3.32E-03 | 1.93E-02 |
| FFG.R | THA.R | 7.78E-08 | 3.55E-03 | 5.37 | 6.19E-04 | 2.89E-01 | 5.40E-03 | 2.95E-07 | 4.14E-02 | 3.79E-02 |
| ORBinf.L | PCUN.L | 1.20E-07 | 1.68E-01 | 5.29 | 8.04E-05 | 6.91E-12 | 1.13E-01 | 7.46E-01 | 2.13E-01 | 7.77E-01 |
| SMA.R | PUT.R | 1.25E-07 | 7.78E-03 | -5.29 | 6.00E-03 | 3.68E-04 | 3.15E-03 | 2.74E-05 | 8.54E-01 | 4.66E-01 |
| ROL.L | THA.L | 1.60E-07 | 1.01E-03 | 5.24 | 3.95E-02 | 7.93E-04 | 1.00E-02 | 1.19E-04 | 7.37E-01 | 2.65E-02 |
| ORBinf.R | SMG.L | 1.72E-07 | 2.64E-05 | -5.23 | 1.71E-01 | 6.72E-03 | 2.10E-01 | 1.39E-03 | 7.85E-02 | 1.50E-03 |
| IFGtriang.L | PCUN.L | 1.74E-07 | 5.31E-05 | 5.23 | 2.39E-01 | 4.48E-06 | 6.08E-02 | 1.18E-01 | 1.75E-02 | 7.66E-01 |
| PreCG.L | THA.R | 1.75E-07 | 1.37E-03 | 5.22 | 5.13E-03 | 3.60E-01 | 5.54E-05 | 2.44E-03 | 4.23E-03 | 5.19E-02 |
| IFGoperc.R | IFGtriang.L | 1.97E-07 | 2.02E-04 | -5.20 | 1.87E-01 | 8.10E-07 | 8.84E-01 | 1.15E-01 | 1.11E-02 | 3.53E-01 |
| SFGmed.R | THA.L | 2.12E-07 | 2.13E-03 | -5.19 | 6.77E-03 | 4.81E-02 | 2.67E-04 | 3.82E-05 | 6.21E-01 | 4.65E-02 |
| FFG.L | TPOmid.R | 2.29E-07 | 4.97E-05 | -5.17 | 6.85E-01 | 1.27E-04 | 9.80E-02 | 1.00E-02 | 5.18E-02 | 5.11E-01 |
| FFG.L | THA.R | 2.42E-07 | 7.88E-03 | 5.16 | 4.42E-04 | 3.64E-01 | 3.55E-02 | 1.35E-07 | 3.27E-03 | 2.75E-01 |
| MFG.L | THA.L | 2.57E-07 | 8.71E-03 | -5.15 | 3.49E-05 | 1.79E-01 | 6.51E-04 | 1.33E-06 | 8.39E-01 | 3.03E-03 |
| ORBinf.R | SMG.R | 2.86E-07 | 1.70E-04 | -5.13 | 8.28E-02 | 5.14E-02 | 1.39E-01 | 6.82E-05 | 2.22E-02 | 2.00E-02 |
| IFGoperc.R | IPL.R | 3.00E-07 | 4.48E-07 | -5.12 | 3.89E-01 | 1.43E-05 | 4.94E-01 | 7.40E-02 | 9.92E-02 | 2.67E-02 |
| IFGoperc.R | IFGtriang.R | 3.87E-07 | 1.23E-06 | -5.08 | 3.72E-01 | 3.04E-05 | 2.32E-01 | 2.54E-01 | 7.39E-02 | 1.50E-02 |
| IFGoperc.L | PCUN.L | 4.95E-07 | 5.79E-04 | 5.03 | 1.62E-01 | 1.46E-05 | 6.03E-02 | 2.40E-02 | 8.28E-02 | 6.43E-01 |
| ORBinf.R | PUT.R | 5.06E-07 | 2.56E-05 | -5.02 | 5.64E-01 | 5.15E-05 | 6.69E-01 | 2.89E-02 | 2.34E-02 | 1.64E-01 |
| SMG.R | PUT.R | 7.07E-07 | 4.35E-03 | -4.96 | 4.17E-03 | 1.95E-01 | 2.17E-05 | 2.31E-04 | 1.10E-01 | 3.78E-01 |
| THA.R | MTG.L | 7.82E-07 | 1.26E-02 | 4.94 | 8.09E-05 | 9.44E-02 | 2.18E-05 | 1.56E-04 | 6.87E-01 | 5.56E-03 |
| SPG.L | THA.R | 8.67E-07 | 6.32E-06 | 4.92 | 3.16E-01 | 4.39E-02 | 6.03E-03 | 8.71E-02 | 8.01E-04 | 1.64E-01 |
| PoCG.L | MTG.L | 8.80E-07 | 3.87E-03 | -4.92 | 7.39E-04 | 2.36E-01 | 1.36E-04 | 6.53E-02 | 1.47E-01 | 1.59E-05 |
| MFG.L | THA.R | 9.60E-07 | 1.66E-02 | -4.90 | 1.02E-06 | 9.05E-01 | 5.50E-04 | 4.23E-08 | 3.59E-01 | 6.21E-03 |
| ORBinf.L | PCUN.R | 1.05E-06 | 3.88E-01 | 4.88 | 9.39E-08 | 1.13E-13 | 1.13E-01 | 8.28E-02 | 5.67E-01 | 9.58E-01 |
| PreCG.L | THA.L | 1.09E-06 | 6.61E-03 | 4.87 | 8.58E-04 | 6.89E-01 | 1.05E-03 | 1.54E-04 | 7.60E-04 | 2.76E-01 |
| THA.L | MTG.L | 1.15E-06 | 3.12E-02 | 4.86 | 5.62E-06 | 1.66E-02 | 2.48E-05 | 9.49E-05 | 1.09E-01 | 9.80E-03 |
| PHG.R | THA.R | 1.41E-06 | 1.37E-04 | 4.82 | 1.36E-01 | 1.75E-01 | 2.68E-03 | 6.83E-03 | 1.13E-03 | 3.69E-01 |
| ROL.L | OLF.R | 1.78E-06 | 8.20E-05 | -4.78 | 1.55E-01 | 8.30E-02 | 6.77E-02 | 3.30E-03 | 5.43E-02 | 1.91E-03 |
| PCL.R | THA.R | 1.91E-06 | 1.70E-04 | 4.76 | 1.03E-01 | 2.89E-02 | 6.36E-03 | 3.32E-02 | 4.77E-01 | 1.61E-04 |
| ROL.R | THA.R | 2.02E-06 | 4.79E-03 | 4.75 | 4.05E-03 | 1.82E-01 | 1.68E-03 | 1.09E-05 | 6.52E-01 | 2.56E-02 |
| IFGoperc.L | IFGtriang.L | 2.47E-06 | 1.31E-02 | -4.71 | 4.41E-02 | 5.81E-05 | 1.58E-01 | 2.40E-01 | 8.78E-04 | 2.96E-01 |
| PCL.R | THA.L | 2.57E-06 | 1.24E-05 | 4.70 | 3.23E-01 | 5.08E-02 | 3.96E-03 | 9.17E-03 | 1.63E-01 | 2.70E-02 |
| SFGmed.L | THA.L | 2.59E-06 | 8.32E-06 | -4.70 | 3.63E-01 | 3.58E-02 | 2.76E-02 | 2.24E-03 | 1.40E-01 | 4.03E-02 |
| IFGoperc.L | TPOmid.R | 2.90E-06 | 8.00E-02 | 4.68 | 9.06E-03 | 4.06E-08 | 6.11E-01 | 3.91E-01 | 4.97E-02 | 4.97E-01 |
| OLF.R | INS.R | 3.52E-06 | 1.80E-05 | -4.64 | 3.59E-01 | 7.57E-04 | 1.49E-01 | 1.79E-03 | 4.25E-01 | 3.04E-01 |
| ROL.R | THA.L | 3.61E-06 | 2.10E-03 | 4.63 | 2.75E-02 | 5.47E-02 | 9.28E-03 | 1.47E-04 | 8.38E-01 | 1.29E-02 |
| ORBinf.L | ROL.R | 3.68E-06 | 1.51E-03 | -4.63 | 9.89E-01 | 1.33E-03 | 2.11E-01 | 1.00E-01 | 3.55E-02 | 8.08E-02 |
| MFG.R' | IFGoperc.R | 3.90E-06 | 3.10E-06 | -4.62 | 4.40E-01 | 5.70E-04 | 5.73E-01 | 3.03E-01 | 3.62E-02 | 2.89E-03 |
| IOG.L | TPOmid.L | 3.99E-06 | 8.18E-03 | -4.61 | 5.14E-03 | 1.14E-01 | 1.20E-03 | 3.35E-05 | 3.56E-01 | 4.42E-01 |
| MFG.R | THA.L | 4.50E-06 | 7.91E-02 | -4.59 | 4.76E-12 | 8.08E-01 | 1.62E-07 | 9.74E-10 | 2.76E-01 | 2.79E-01 |
| IPL.R | PAL.R | 4.70E-06 | 4.82E-03 | -4.58 | 7.39E-02 | 4.28E-04 | 1.78E-02 | 2.59E-03 | 8.92E-01 | 4.41E-01 |
| ORBinf.L | PCG.L | 4.74E-06 | 9.20E-02 | 4.58 | 4.77E-03 | 5.11E-09 | 5.00E-01 | 8.81E-01 | 4.46E-01 | 2.55E-01 |
| DCG.R | PUT.R | 4.84E-06 | 6.05E-02 | -4.57 | 2.13E-05 | 1.85E-02 | 2.07E-03 | 3.90E-07 | 8.58E-01 | 6.19E-01 |
| PAL.R | THA.R | 5.37E-06 | 4.71E-06 | -4.55 | 4.14E-01 | 3.37E-03 | 2.95E-01 | 4.56E-04 | 1.56E-01 | 4.51E-01 |
| SMG.R | PAL.R | 6.06E-06 | 2.38E-02 | -4.52 | 2.14E-04 | 1.87E-01 | 4.78E-05 | 1.49E-05 | 8.40E-01 | 4.66E-01 |
| FFG.R | THA.L | 6.62E-06 | 1.03E-02 | 4.51 | 2.21E-03 | 4.80E-01 | 3.02E-03 | 4.62E-06 | 8.46E-02 | 3.78E-01 |
| THA.L | HES.L | 6.68E-06 | 2.10E-02 | 4.50 | 2.53E-04 | 4.20E-02 | 1.16E-04 | 1.79E-03 | 2.44E-01 | 2.15E-03 |
| SMG.L | PUT.R | 7.06E-06 | 2.97E-03 | -4.49 | 1.82E-02 | 3.38E-01 | 3.67E-03 | 9.89E-05 | 1.18E-01 | 1.17E-01 |
| THA.L | THA.R | 8.00E-06 | 9.75E-03 | -4.47 | 1.66E-03 | 9.38E-01 | 3.70E-02 | 1.74E-05 | 2.28E-04 | 2.53E-01 |
| REC.R | TPOmid.R | 8.24E-06 | 6.72E-02 | -4.46 | 2.01E-03 | 1.44E-04 | 6.12E-01 | 6.17E-01 | 8.24E-06 | 6.47E-01 |
| THA.L | STG.L | 8.41E-06 | 2.33E-02 | 4.45 | 1.77E-04 | 4.46E-02 | 2.77E-04 | 8.54E-04 | 2.26E-01 | 2.54E-03 |
| REC.R | ANG.R | 8.54E-06 | 5.62E-04 | 4.45 | 2.39E-01 | 1.73E-05 | 9.95E-01 | 4.25E-01 | 4.93E-03 | 5.31E-01 |
| ORBinf.R | ROL.R | 9.02E-06 | 6.24E-05 | -4.44 | 5.48E-01 | 1.13E-02 | 7.69E-02 | 3.88E-01 | 4.42E-04 | 3.63E-01 |
| INS.R | AMYG.R | 9.80E-06 | 4.23E-04 | -4.42 | 7.14E-01 | 1.22E-02 | 8.42E-03 | 2.29E-01 | 1.88E-02 | 3.32E-01 |
| ORBinf.R | PCUN.L | 9.81E-06 | 3.30E-02 | 4.42 | 2.01E-02 | 7.80E-07 | 1.40E-01 | 5.25E-01 | 2.96E-01 | 8.06E-02 |
| SFGmed.R | THA.R | 1.04E-05 | 1.44E-02 | -4.41 | 7.89E-04 | 3.11E-01 | 1.70E-04 | 3.83E-05 | 8.44E-01 | 8.89E-02 |
| MFG.R | SOG.L | 1.11E-05 | 5.64E-03 | 4.39 | 2.70E-02 | 2.09E-01 | 1.94E-03 | 8.48E-04 | 2.15E-02 | 9.64E-01 |
| PoCG.L | PoCG.R | 1.12E-05 | 3.99E-05 | -4.39 | 3.32E-01 | 2.06E-02 | 5.91E-01 | 8.81E-04 | 6.66E-02 | 6.13E-02 |
| DCG.R | AMYG.R | 1.19E-05 | 3.20E-02 | -4.38 | 1.19E-03 | 1.18E-02 | 3.73E-03 | 2.55E-05 | 5.23E-01 | 5.44E-01 |
| MFG.R | PAL.R | 1.24E-05 | 7.67E-02 | -4.37 | 1.61E-04 | 1.14E-03 | 3.21E-02 | 4.28E-06 | 2.89E-01 | 9.34E-01 |

P value of FE*: p value of fixed-effects model.

P value of RE*: p value of random-effects model.

P value of Hete.*: p value of Cochran’s Q test for heterogeneity.

Z score*: negative z indicates weaker functional connectivity and positive z indicates stronger FC in schizophrenia patients than controls.

**Table S5. The pattern of links connect with thalamus**

|  | **Prefontal** | **Motor** | **Parietal** | **Occipital** | **Temporal** | **Subcortical** |
| --- | --- | --- | --- | --- | --- | --- |
| **Total links** | 10 | 8 | 5 | 0 | 8 | 35 |
| **Increased links** | 3 | 8 | 5 | 0 | 8 | 24 |
| **Decreased links** | 7 | 0 | 0 | 0 | 0 | 11 |

Total links: the number of links involving thalamus.

Increased links: the number of links, involved with thalamus, increase in patient group.

Decreased links: the number of links, involved with thalamus, decrease in patient group.

**Table S6.** Correlations between the other functional connectivity links (except thalamus) and the symptom severity scores (PANSS) and illness duration.

| **Links** | | **Positive scale** | | **Negative scale** | | **General scale** | | **Illness duration** | |
| --- | --- | --- | --- | --- | --- | --- | --- | --- | --- |
|  |  | **Correlation** | **P value** | **Correlation** | **P value** | **Correlation** | **P value** | **Correlation** | **P value** |
| **IFGoperc.R** | **TPOmid.R** | 0.0853 | 0.1066 | -0.0079 | 0.8814 | 0.0992 | 0.0584 | 0.0399 | 0.4385 |
| **IFGtriang.R** | **TPOmid.R** | 0.0194 | 0.7189 | -0.0083 | 0.8685 | 0.0043 | 0.9357 | 0.0836 | 0.1121 |
| **INS.L** | **PUT.R** | -0.0045 | 0.9337 | -0.0596 | 0.2551 | 0.0233 | 0.6534 | -0.0791 | 0.1254 |
| **ORBinf.R** | **INS.R** | 0.0034 | 0.9489 | -0.1025 | 0.0517 | 0.0158 | 0.7640 | 0.0304 | 0.5675 |
| **ORBinf.R** | **ROL.L** | 0.0243 | 0.6454 | -0.0898 | 0.0889 | 0.0491 | 0.3514 | 0.0750 | 0.1564 |
| **ORBinf.R** | **INS.L** | 0.0690 | 0.1927 | **-0.1350** | **0.0102** | 0.0263 | 0.6147 | -0.0043 | 0.9356 |
| **ORBinf.L** | **PCUN.L** | 0.0669 | 0.1990 | -0.0157 | 0.7674 | 0.0792 | 0.1338 | 0.0501 | 0.3258 |
| **SMA.R** | **PUT.R** | -0.0075 | 0.8889 | -0.0212 | 0.6906 | 0.0220 | 0.6700 | -0.0662 | 0.2019 |
| **ORBinf.R** | **SMG.L** | -0.0926 | 0.0801 | -0.0750 | 0.1549 | -0.0513 | 0.3322 | 0.0520 | 0.3262 |
| **IFGtriang.L** | **PCUN.L** | -0.0021 | 0.9741 | -0.0045 | 0.9299 | -0.0149 | 0.7765 | 0.0459 | 0.3852 |
| **IFGoperc.R** | **IFGtriang.L** | 0.0305 | 0.5672 | -0.0582 | 0.2677 | -0.0369 | 0.4816 | -0.0024 | 0.9632 |
| **FFG.L** | **TPOmid.R** | -0.0545 | 0.2988 | -0.0179 | 0.7359 | 0.0170 | 0.7416 | 0.0213 | 0.6839 |
| **ORBinf.R** | **SMG.R** | -0.0695 | 0.1879 | -0.0400 | 0.4423 | -0.0640 | 0.2254 | 0.0274 | 0.6106 |
| **IFGoperc.R** | **IPL.R** | -0.0779 | 0.1362 | 0.0143 | 0.7994 | -0.0500 | 0.3268 | 0.0450 | 0.3955 |
| **IFGoperc.R** | **IFGtriang.R** | **0.1089** | **0.0383** | 0.0243 | 0.6434 | 0.0589 | 0.2672 | -0.0494 | 0.3415 |
| **IFGoperc.L** | **PCUN.L** | 0.0706 | 0.1668 | 0.0360 | 0.5055 | 0.0263 | 0.6238 | 0.0681 | 0.1956 |
| **ORBinf.R** | **PUT.R** | 0.0366 | 0.4866 | -0.0740 | 0.1516 | 0.0843 | 0.1093 | 0.0161 | 0.7664 |
| **SMG.R** | **PUT.R** | 0.0716 | 0.1762 | -0.0780 | 0.1410 | -0.0246 | 0.6374 | -0.0013 | 0.9795 |
| **PoCG.L** | **MTG.L** | 0.0212 | 0.6715 | -0.0384 | 0.4684 | 0.0029 | 0.9533 | -0.0087 | 0.8694 |
| **ORBinf.L** | **PCUN.R** | 0.0935 | 0.0743 | -0.0070 | 0.8934 | 0.0708 | 0.1818 | -0.0544 | 0.3235 |
| **ROL.L** | **OLF.R** | -0.0113 | 0.8113 | -0.0419 | 0.4288 | 0.0508 | 0.3294 | -0.0301 | 0.5706 |
| **IFGoperc.L** | **IFGtriang.L** | **-0.1486** | **0.0043** | 0.0358 | 0.5004 | -0.0357 | 0.4945 | -0.0094 | 0.8592 |
| **IFGoperc.L** | **TPOmid.R** | -0.0028 | 0.9567 | -0.0444 | 0.3933 | 0.0237 | 0.6458 | -0.0050 | 0.9246 |
| **OLF.R** | **INS.R** | -0.0056 | 0.9170 | 0.0363 | 0.4741 | 0.1225 | 0.0182 | 0.0057 | 0.8908 |
| **ORBinf.L** | **ROL.R** | -0.0157 | 0.7668 | -0.0463 | 0.3835 | -0.0345 | 0.5157 | 0.0433 | 0.4134 |
| **MFG.R** | **IFGoperc.R** | 0.0838 | 0.1137 | 0.0738 | 0.1616 | -0.0068 | 0.8927 | -0.0197 | 0.7104 |
| **IOG.L** | **TPOmid.L** | -0.0497 | 0.3487 | -0.0382 | 0.4684 | 0.0352 | 0.5192 | -0.0488 | 0.3545 |
| **IPL.R** | **PAL.R** | 0.0593 | 0.2588 | -0.0297 | 0.5744 | -0.0026 | 0.9546 | 0.0413 | 0.4354 |
| **ORBinf.L** | **PCG.L** | -0.0286 | 0.5838 | 0.0146 | 0.7835 | -0.0026 | 0.9495 | -0.0523 | 0.3049 |
| **DCG.R** | **PUT.R** | 0.0990 | 0.0574 | -0.0044 | 0.9276 | 0.0189 | 0.7224 | **-0.1362** | **0.0075** |
| **SMG.R** | **PAL.R** | **0.1122** | **0.0283** | -0.0465 | 0.3796 | 0.0000 | 0.9963 | -0.0012 | 0.9817 |
| **SMG.L** | **PUT.R** | 0.0084 | 0.8729 | **-0.1283** | **0.0144** | -0.0464 | 0.3809 | -0.0080 | 0.8650 |
| **REC.R** | **TPOmid.R** | 0.0363 | 0.4881 | 0.0807 | 0.1270 | 0.0719 | 0.1722 | 0.0583 | 0.2586 |
| **REC.R** | **ANG.R** | -0.0832 | 0.1090 | -0.0022 | 0.9663 | -0.0161 | 0.7556 | -0.0910 | 0.0825 |
| **ORBinf.R** | **ROL.R** | -0.0626 | 0.2344 | -0.0680 | 0.1983 | -0.0103 | 0.8388 | 0.0906 | 0.0866 |
| **INS.R** | **AMYG.R** | **0.1155** | **0.0273** | -0.0357 | 0.5111 | **0.1269** | **0.0113** | -0.1018 | 0.0543 |
| **ORBinf.R** | **PCUN.L** | 0.0706 | 0.1832 | -0.0352 | 0.5048 | 0.0964 | 0.0666 | -0.0545 | 0.3102 |
| **MFG.R** | **SOG.L** | -0.0462 | 0.3822 | -0.0143 | 0.7852 | -0.0190 | 0.7198 | 0.0395 | 0.4559 |
| **PoCG.L** | **PoCG.R** | 0.0291 | 0.5836 | -0.1014 | 0.0528 | -0.0407 | 0.4424 | **-0.1217** | **0.0210** |
| **DCG.R** | **AMYG.R** | 0.1433 | 0.0065 | -0.0691 | 0.1825 | 0.0135 | 0.7979 | -0.0929 | 0.0719 |
| **MFG.R** | **PAL.R** | 0.1503 | 0.0040 | 0.0168 | 0.7469 | 0.0501 | 0.3453 | 0.0174 | 0.7364 |

**Table S7.** Results of classification.

|  | **Huaxi** | **COBRE** | **Taiwan** | **Xiangya** | **Nottingham** | **All data** |
| --- | --- | --- | --- | --- | --- | --- |
| **Sensitivity** | 83.23% | 90.57% | 91.30% | 84.34% | 68.75 % | 76.63% |
| **Specificity** | 73.33% | 71.64% | 69.13% | 65.00% | 77.78% | 74.93 % |
| **Accuracy** * | 78.46% | 80.00 % | 80.92% | 76.22% | 73.53% | 75.81% |

* An alternative method to cross-validate may be to use results from one dataset for training and validate the results on another test dataset. But this approach is inherently weak in cases where reliable patterns may not be detected in underpowered datasets, a problem that has particularly affected the neuroimaging studies in schizophrenia. As a result, for SVM we chose the most robust patterns emerging from the combined results and employed a leave-one-out cross-validation approach.

**Table S8.** Comparison of mean head motion. The negative t value indicate the mean motion was higher for patients.

| **Statistic** | | **COBRE** | **Taiwan** | **Xiangya** | **Huaxi** | **Nottingham** | **All data** |
| --- | --- | --- | --- | --- | --- | --- | --- |
| Mean Displacement | T value | 0.3893 | -2.8478 | -2.0841 | -0.5945 | -1.7110 | -1.8509 |
|  | P value | 0.6978 | 0.0051 | 0.0390 | 0.5526 | 0.0918 | 0.0646 |
| Mean Rotation | T value | 0.6735 | -1.8899 | -2.0514 | 0.3452 | -2.0852 | -0.8746 |
|  | P value | 0.5019 | 0.0610 | 0.0421 | 0.7301 | 0.0409 | 0.3821 |

ue indicate the mean motion was ead motion. lysis. 0000000000000000000000000000000000000000000000000000000000000000000000000000**Figure S1.** A) Oxford thalamic connectivity atlas. The thalamic atlas contains 7 thalamic sub-regions, segmented according to their white-matter connectivity to cortical areas. thalamic sub-region 1 connects to primary motor cortex; thalamic sub-region 2 connects to sensory cortex; thalamic sub-region 3 connects to occipital cortex; thalamic sub-region 4 connects to pre-frontal cortex; thalamic sub-region 5 connects to pre-motor cortex; thalamic sub-region 6 connects to posterior parietal cortex; thalamic sub-region 7 connects to temporal cortex. B) Map of the postcentral atlas, with the postcentral gyrus divided into 3 sub-regions ([Brodmann areas](http://en.wikipedia.org/wiki/Brodmann_area) 1, 2 and 3).

**
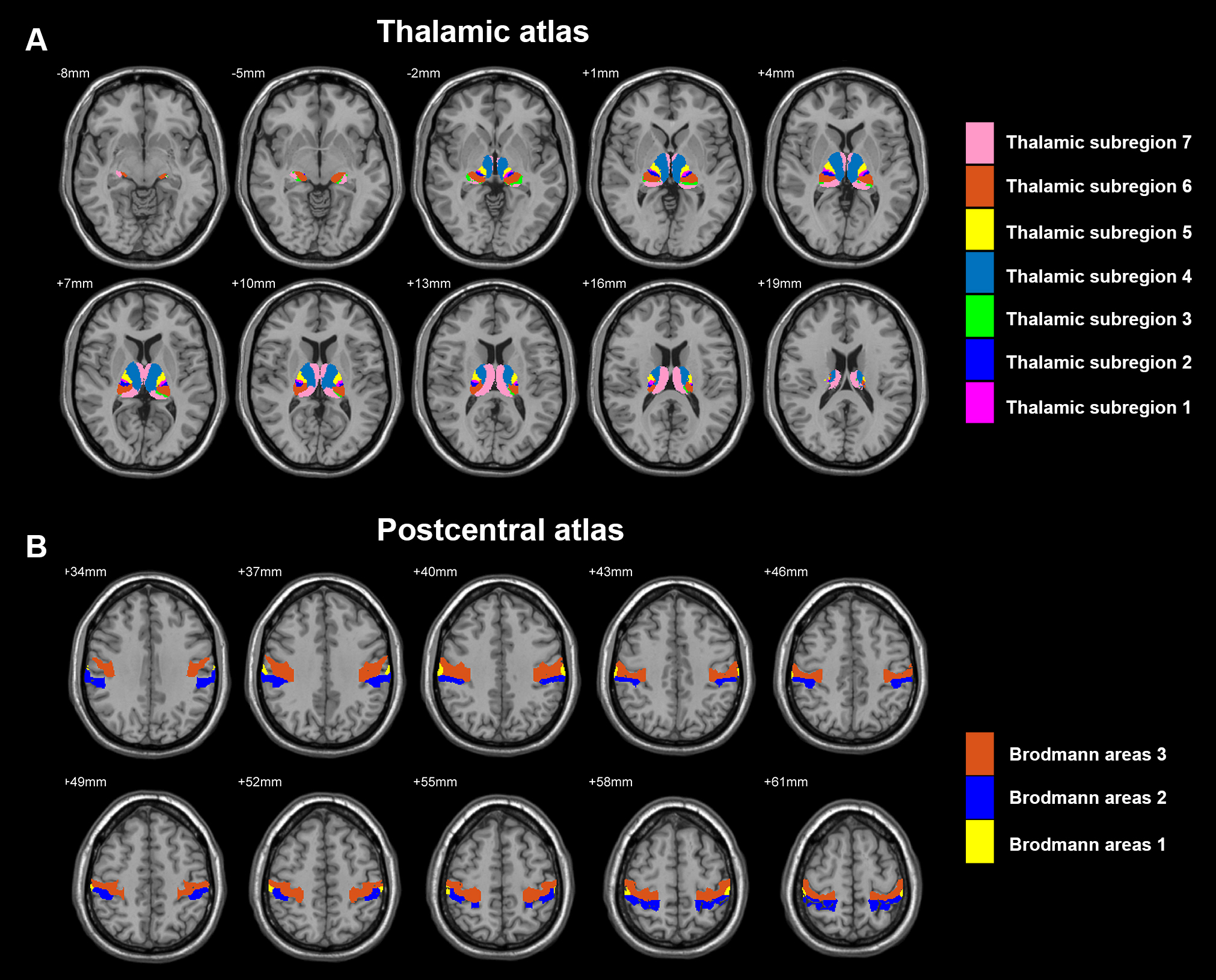
**

**Figure S2.** Map of thalamic sub-regions with functional connectivity pattern differences between the schizophrenia patients and the controls. Blue indicates that schizophrenia subjects had decreased functional connectivity compared with the controls and the yellow/red indicates the opposite. Threshold was set at p < 0.05 (Bonferroni correction). The majority of changes involved thalamic sub-region 4, which is primarily the dorsomedial nucleus connected to pre-frontal cortex in the Oxford thalamic connectivity atlas, and sub-region 5 which is mainly the ventral lateral nucleus connected to pre-motor cortex in Oxford thalamic connectivity atlas. A pattern of increased thalamic functional connectivity with motor and sensory regions are notable; there is also reduced functional connectivity between the right and left thalamus for all sub-regions.


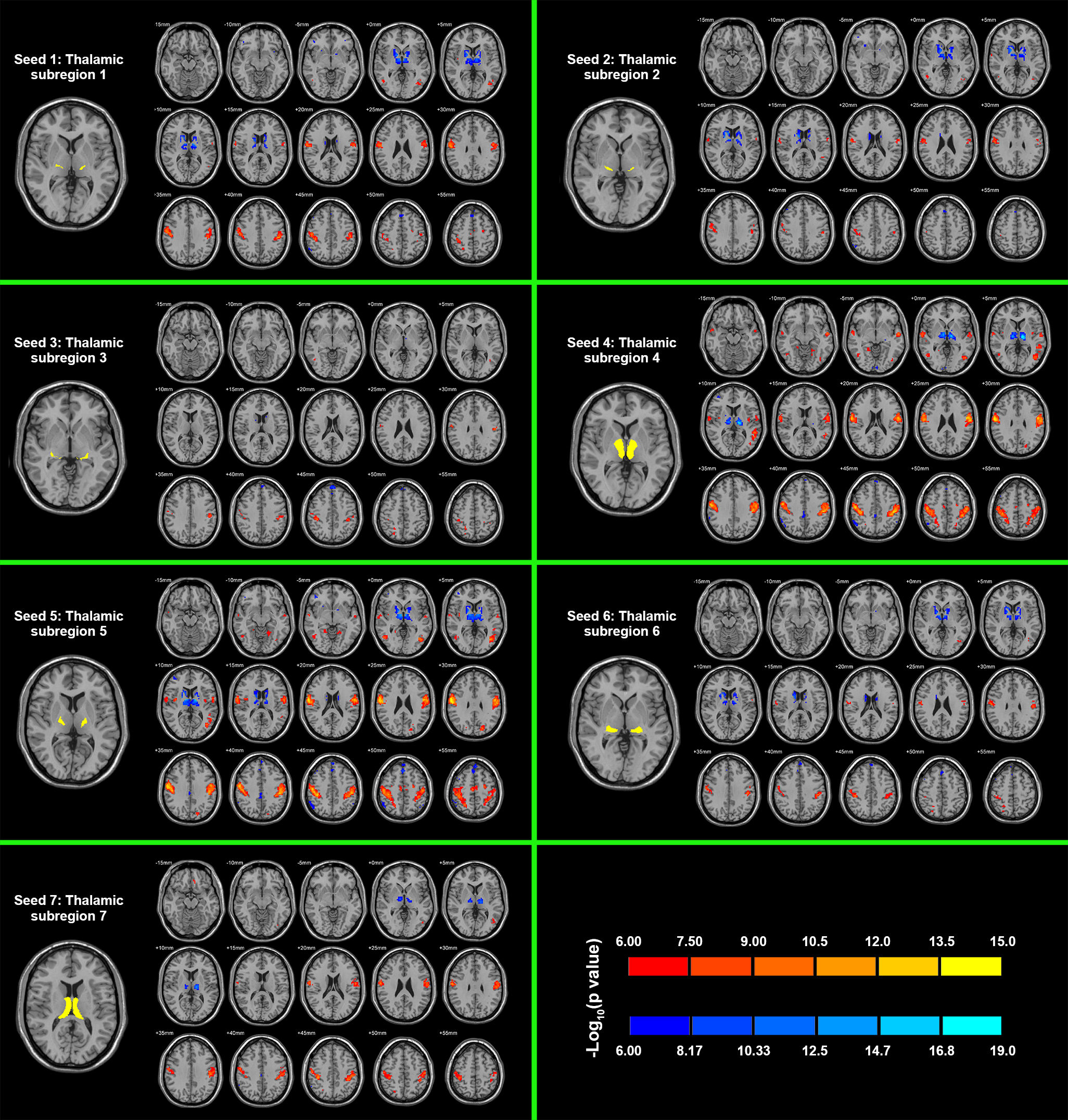


**Figure S3.** Map of differences between the schizophrenia patients and controls in functional connectivity of postcentral sub-regions with the thalamus. Blue indicates that schizophrenia subjects had decreased functional connectivity compared with the controls and the yellow indicates the opposite. Threshold was set at p < 0.05 (Bonferroni correction). Most changes involved the primary somatosensory cortex (Brodmann area 3).


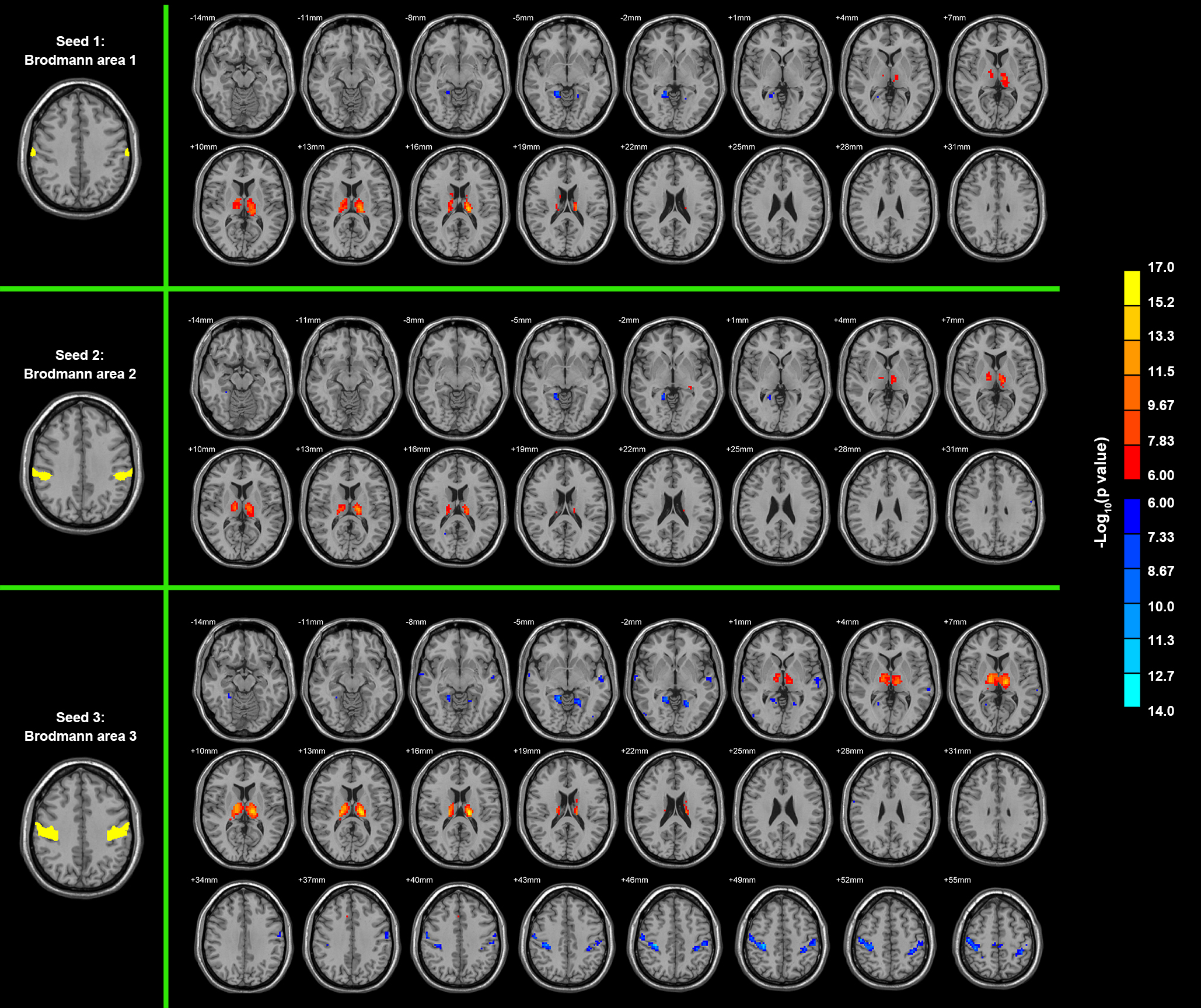


**References**

1 Power, J. D., Barnes, K. A., Snyder, A. Z., Schlaggar, B. L. & Petersen, S. E. Spurious but systematic correlations in functional connectivity MRI networks arise from subject motion. *Neuroimage* **59**, 2142-2154 (2012).

2 Van Dijk, K. R., Sabuncu, M. R. & Buckner, R. L. The influence of head motion on intrinsic functional connectivity MRI. *Neuroimage* **59**, 431-438 (2012).

3 Saad, Z. S. *et al.* Trouble at rest: how correlation patterns and group differences become distorted after global signal regression. *Brain connectivity* **2**, 25-32 (2012).

4 Fox, M. D., Zhang, D., Snyder, A. Z. & Raichle, M. E. The global signal and observed anticorrelated resting state brain networks. *Journal of neurophysiology* **101**, 3270-3283 (2009).

5 Hayasaka, S. Functional connectivity networks with and without global signal correction. *Frontiers in human neuroscience* **7** (2013).

6 Yan, C.-G. *et al.* A comprehensive assessment of regional variation in the impact of head micromovements on functional connectomics. *Neuroimage* **76**, 183-201 (2013).

7 Murphy, K., Birn, R. M., Handwerker, D. A., Jones, T. B. & Bandettini, P. A. The impact of global signal regression on resting state correlations: are anti-correlated networks introduced? *Neuroimage* **44**, 893-905 (2009).

8 Pettersson-Yeo, W., Allen, P., Benetti, S., McGuire, P. & Mechelli, A. Dysconnectivity in schizophrenia: where are we now? *Neuroscience & Biobehavioral Reviews* **35**, 1110-1124 (2011).

9 Wang, K. *et al.* Altered functional connectivity in early Alzheimer's disease: A resting‐state fMRI study. *Human brain mapping* **28**, 967-978 (2007).

10 Bai, F. *et al.* Abnormal whole-brain functional connection in amnestic mild cognitive impairment patients. *Behavioural brain research* **216**, 666-672 (2011).
